# Supplementary figures and images for: Social support and ideal cardiovascular health in urban Jamaica: A cross-sectional study
Source: PLOS Glob Public Health. 2024 Jul 30;4(7):e0003466. doi: 10.1371/journal.pgph.0003466 (PMC11288424; doi:10.1371/journal.pgph.0003466)

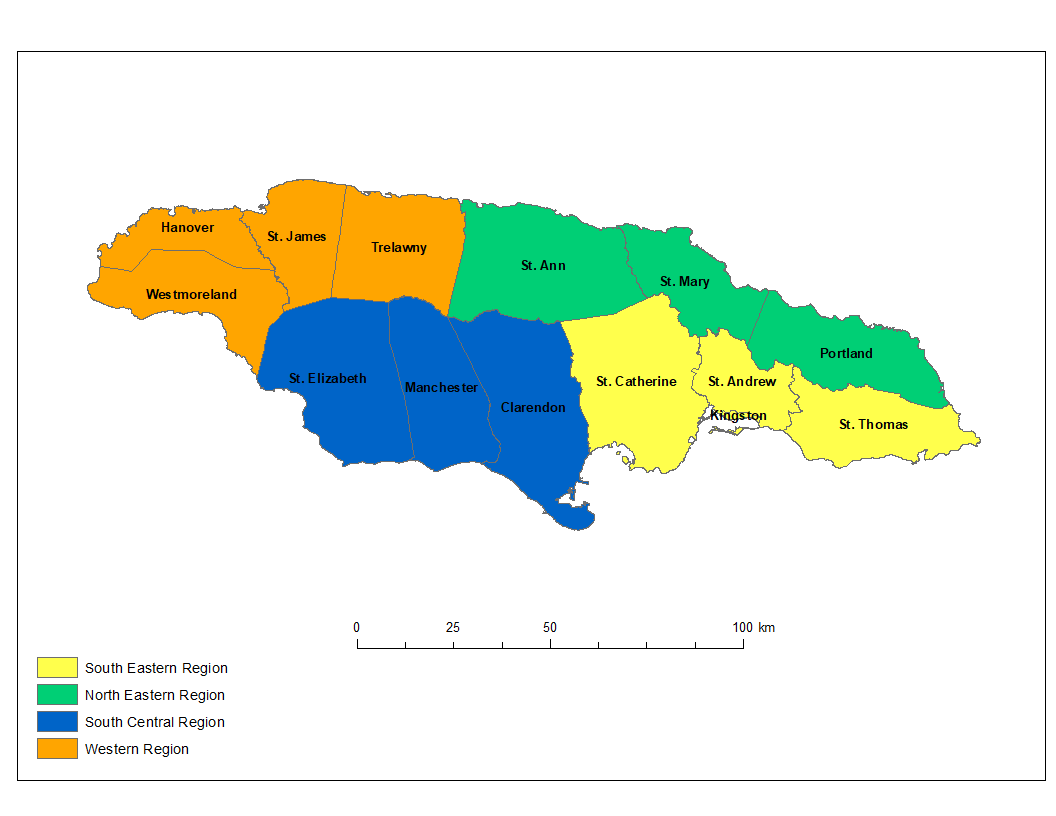

Supplement: S1 Fig — The study was conducted in communities selected from Jamaica’s Southeast Health Region (yellow-coloured parishes on the map). Base layer map obtained from Humanitarian Data Exchange (web link: https://data.humdata.org/dataset/cod-ab-jam); source map is licensed under a Creative Commons Attribution 4.0 International license. (TIF) [file pgph.0003466.s002.tif]
