# Supplementary material for: Social support and ideal cardiovascular health in urban Jamaica: A cross-sectional study
Source: PLOS Glob Public Health. 2024 Jul 30;4(7):e0003466. doi: 10.1371/journal.pgph.0003466 (PMC11288424; doi:10.1371/journal.pgph.0003466)
Supplement: S1 Table — (DOCX) [file pgph.0003466.s003.docx]

**Table S1: Mean values for participant characteristics by sex (estimates weighted for survey design; no imputation)**

| Characteristic | Male  n= 279 | Female  n= 562 | Total  N=841 | p-value for sex difference |
| --- | --- | --- | --- | --- |
| *Mean for Continuous Variables* | Mean ± SE | Mean ± SE | Mean ± SE |  |
| Age (years) | 38.5 ± 0.1 | 38.1 ± 0.9 | 38.3±0.1 | 0.015 |
| Height (cm) | 173.4 ± 0.6 | 162.8 ± 0.6 | 167.9±0.5 | <0.001 |
| Weight (kg) | 77.4 ± 1.1 | 78.8 ± 1.2 | 78.1±0.8 | 0.383 |
| Body Mass Index (kg/m2) | 25.6 ±0.4 | 29.6 ± 0.4 | 27.7±0.3 | <0.001 |
| Systolic Blood Pressure (mmHg) | 127.6 ±0.8 | 121.7 ±1.4 | 124.5±0.9 | <0.001 |
| Diastolic Blood Pressure (mmHg) | 81.6 ±0.6 | 80.8 ±0.9 | 81.2±0.6 | 0.399 |
| Fasting Glucose (mmol/L) | 5.8 ± 0.4 | 5.5 ±0.1 | 5.6±0.2 | 0.342 |
| Total Cholesterol (mmol/L) | 4.2 ± 0.1 | 4.2 ± 0.1 | 4.2±0.1 | 0.854 |
| ICH Score | 3.7 ± 0.1 | 3.5 ± 0.1 | 3.6 ± 0.1 | 0.156 |
| Social Support Score (PCA) | 0.22 ± 0.1 | -0.15 ± 0.1 | 0.0 ± 0.1 | 0.004 |
|  |  |  |  |  |
| Categorical Variables | **% ± SE** | **% ± SE** | **% ± SE** |  |
| Education |  |  |  | 0.851 |
| Less than High School | 12.4±1.2 | 11.2±1.6 | 11.8±1.1 |  |
| High School | 53.5±3.6 | 53.3±3.7 | 53.4±3.1 |  |
| More than High School | 34.1±4.0 | 35.5±4.1 | 34.8±3.4 |  |
| Median Land Value |  |  |  | 0.264 |
| Lower Tertile | 46.4±7.2 | 50.6±7.9 | 48.6±7.4 |  |
| Middle Tertile | 38.7±6.5 | 34.4±6.3 | 36.5±6.2 |  |
| Upper Tertile | 14.9±4.8 | 15.1±5.3 | 15.0±4.9 |  |
| Community Poverty |  |  |  | 0.260 |
| Lower Tertile | 46.1±8.1 | 40.4±9.0 | 43.1±8.4 |  |
| Middle Tertile | 21.4±6.0 | 22.0±6.4 | 21.7±6.1 |  |
| Lower Tertile | 32.5±5.6 | 37.5±7.6 | 35.1±6.5 |  |

*ICH – Ideal Cardiovascular Health; PCA- Principal Component Analysis

ICH Score = sum of number of individual ICH components

Social Support Score = PCA derived score from number of friends, number of friends willing to give advice and number of willing to provide loans.

Median Land Value – median property value for community based on data from National Land Agency

Community poverty – based on data from Jamaica Poverty May 2019
