# Supplementary material for: Social support and ideal cardiovascular health in urban Jamaica: A cross-sectional study
Source: PLOS Glob Public Health. 2024 Jul 30;4(7):e0003466. doi: 10.1371/journal.pgph.0003466 (PMC11288424; doi:10.1371/journal.pgph.0003466)
Supplement: S2 Table — (DOCX) [file pgph.0003466.s004.docx]

**Table S2A: Population age-sex numbers and proportions for urban Jamaica**

| Age | Population total - Urban Males  n | Population total - Urban Females  n | Percentage of Males in age groups  % | Percentage of Females in age groups  % | Percentage of Total in age groups  % |
| --- | --- | --- | --- | --- | --- |
| 15-24 | 140865 | 143549 | 27.2 | 25.0 | 26.0 |
| 25-34 | 111432 | 124757 | 21.5 | 21.7 | 21.6 |
| 35-44 | 93785 | 106845 | 18.1 | 18.6 | 18.4 |
| 45-54 | 75726 | 86588 | 14.6 | 15.1 | 14.9 |
| 55-64 | 48569 | 52389 | 9.4 | 9.1 | 9.2 |
| 65-74 | 27476 | 31060 | 5.3 | 5.4 | 5.4 |
| 75+ | 19703 | 29171 | 3.8 | 5.1 | 4.5 |
| Total | 517556 | 574359 | 100 | 100 | 100 |

**Table S2B: Sample age-sex numbers and proportions, weighted age-sex numbers, and proportions**

| **Age Groups** | **Sample total - Urban Males**  **n** | **Sample total - Urban Females**  **n** | **Percentage of Males in sample age groups**  **%** | **Percentage of Females in sample age groups**  **%** | **Percentage of Total in sample age groups**  **%** | **Weighted percentages of Urban Males in age groups**  **%** | **Weighted percentages of Urban Females in age groups**  **%** | **Weighted percentages of Urban residents in age groups**  **%** |
| --- | --- | --- | --- | --- | --- | --- | --- | --- |
| **15-24** | 50 | 68 | 17.6 | 12.1 | 13.9 | 27.2 | 25.7 | 26.4 |
| **25-34** | 43 | 82 | 15.4 | 14.6 | 14.9 | 21.2 | 22.2 | 21.7 |
| **35-44** | 36 | 92 | 12.9 | 16.0 | 15.0 | 18.1 | 19.2 | 18.6 |
| **45-54** | 52 | 109 | 18.6 | 19.2 | 19.0 | 15.0 | 15.9 | 15.4 |
| **55-64** | 39 | 97 | 13.6 | 17.3 | 16.1 | 9.5 | 9.2 | 9.3 |
| **65-74** | 35 | 77 | 12.5 | 13.5 | 13.2 | 5.3 | 5.4 | 5.3 |
| **75+** | 27 | 42 | 9.3 | 7.3 | 8.0 | 3.7 | 2.6 | 3.1 |
| **Total** | 282 | 567 | 100 | 100 | 100 | 100 | 100 | 100 |
