# Supplementary material for: Social support and ideal cardiovascular health in urban Jamaica: A cross-sectional study
Source: PLOS Glob Public Health. 2024 Jul 30;4(7):e0003466. doi: 10.1371/journal.pgph.0003466 (PMC11288424; doi:10.1371/journal.pgph.0003466)
Supplement: S3 Table — (DOCX) [file pgph.0003466.s005.docx]

**Table S3: Prevalence of ICH-5 by Social Support Score Tertiles and by Socioeconomic Status (SES) Categories**

| SES Variable | Low | Middle | High | P-value for difference in proportions |
| --- | --- | --- | --- | --- |
| **Males and Females** |  |  |  |  |
| Social Support Score^1^ | 33.0 | 22.6 | 23.7 | 0.121 |
| Education Level^2^ | 16.0 | 29.1 | 26.5 | 0.046 |
| Median Land Value | 30.9 | 19.6 | 29.9 | 0.020 |
| Poverty^3^ | 22.3 | 23.1 | 34.2 | 0.056 |
|  |  |  |  |  |
|  |  |  |  |  |
| **Males** |  |  |  |  |
| Social Support Score | 44.3 | 10.4 | 20.8 | <0.001 |
| Education Level | 15.8 | 31.8 | 19.7 | 0.004 |
| Median Land Value | 31.4 | 17.3 | 29.7 | 0.041 |
| Poverty | 20.6 | 23.5 | 34.2 | 0.065 |
|  |  |  |  |  |
| **Females** |  |  |  |  |
| Social Support Score | 23.8 | 32.7 | 26.9 | 0.448 |
| Education Level | 16.2 | 26.6 | 32.5 | 0.087 |
| Median Land Value | 30.5 | 22.0 | 30.1 | 0.157 |
| Poverty | 24.0 | 22.6 | 34.2 | 0.177 |

^1^Social support score, median land value and poverty are categorized into tertiles.

^2^Education is categorized as less the high school, high school and more than high school.

^3^Poverty is categorized as proportion of persons experiencing poverty in a community so that higher categories indicate lower socioeconomic status.
