# Supplementary material for: Social support and ideal cardiovascular health in urban Jamaica: A cross-sectional study
Source: PLOS Glob Public Health. 2024 Jul 30;4(7):e0003466. doi: 10.1371/journal.pgph.0003466 (PMC11288424; doi:10.1371/journal.pgph.0003466)
Supplement: S4 Table — (DOCX) [file pgph.0003466.s006.docx]

**Table S4: Mean Social Support Score (from PCA) by Socioeconomic Status (SES) Categories**

| SES Variable | Low  Mean | Middle  Mean | High  Mean | P-value for difference in means |
| --- | --- | --- | --- | --- |
| Education Level | -0.18 | -0.16 | 0.43 | <0.001 |
| Median Land Value | -0.13 | 0.28 | 0.00 | <0.001 |
| Poverty | 0.03 | 0.39 | -0.17 | 0.016 |
