# Supplementary material for: Social support and ideal cardiovascular health in urban Jamaica: A cross-sectional study
Source: PLOS Glob Public Health. 2024 Jul 30;4(7):e0003466. doi: 10.1371/journal.pgph.0003466 (PMC11288424; doi:10.1371/journal.pgph.0003466)
Supplement: S5 Table — (DOCX) [file pgph.0003466.s007.docx]

**Table S5: Prevalence of Ideal Cardiovascular Health (ICH) Characteristics by Socioeconomic Status (SES) Level**

| **ICH Characteristic** | **SES Category** | | | **P-value for difference in means** |
| --- | --- | --- | --- | --- |
| **Education** | **Less than High School** | **High School** | **More than High School** |  |
| Normal BMI | 31 (22 – 40) | 40 (33 – 46) | 31 (23 – 39) | 0.286 |
| Non-smoker | 83 (73 – 92) | 77 (72 – 82) | 87 (81 – 94) | 0.076 |
| Normal glucose | 45 (35 – 54) | 68 (62 – 74) | 74 (64 – 86) | <0.001 |
| Normal blood pressure | 14 (6 – 22) | 38 (32 – 44) | 36 (27 – 45) | 0.004 |
| Adequate physical activity | 45 (35 – 54) | 40 (34 – 46) | 41 (33 – 49) | 0.652 |
| Healthy diet | 24 (17 – 31) | 19 (14 – 24) | 17 (12 – 22) | 0.248 |
| Normal Cholesterol | 64 (53 – 74) | 83 (79 – 88) | 76 (68 – 84) | 0.005 |
|  |  |  |  |  |
| **Median Land Value** | **Lower Tertile** | **Middle Tertile** | **Upper Tertile** |  |
| Normal BMI | 38 (33 – 44) | 29 (23 – 35) | 43 (36 – 50) | 0.021 |
| Non-smoker | 77 (72 – 83) | 86 (79 – 93) | 82 (74 – 90) | 0.230 |
| Normal glucose | 71 (66 – 77) | 64 (51 – 77) | 64 (56 – 72) | 0.260 |
| Normal blood pressure | 34 (29 – 40) | 35 (25 – 45) | 33 (15 – 51) | 0.969 |
| Adequate physical activity | 40 (33 – 47) | 46 (39 – 53) | 29 (21 – 37) | 0.012 |
| Healthy diet | 16 (11 – 20) | 21 (15 – 27) | 26 (16 – 35) | 0.157 |
| Normal cholesterol | 87 (84 – 90) | 69 (62 – 76) | 74 (63 – 85) | <0.001 |
|  |  |  |  |  |
| **Community Poverty** | **Lower Tertile** | **Middle Tertile** | **Upper Tertile** |  |
| Normal BMI | 35 (30 – 40) | 30 (24 – 36) | 40 (33 – 47) | 0.060 |
| Non-smoker | 82 (78 – 86) | 88 (79 – 97) | 76 (69 – 83) | 0.156 |
| Normal glucose | 61 (51 – 71) | 70 (55 – 84) | 74 (70 – 79) | 0.039 |
| Normal blood pressure | 32 (23 – 41) | 39 (29 – 49) | 34 (28 – 41) | 0.591 |
| Adequate physical activity | 39 (31 – 47) | 46 (38 – 53) | 39 (32 – 47) | 0.450 |
| Healthy diet | 25 (18 – 31) | 13 (8 – 19) | 16 (10 – 21) | 0.048 |
| Normal cholesterol | 74 (68 – 81) | 71 (59 – 82) | 88 (85 – 92) | <0.001 |
