# Supplementary material for: Social support and ideal cardiovascular health in urban Jamaica: A cross-sectional study
Source: PLOS Glob Public Health. 2024 Jul 30;4(7):e0003466. doi: 10.1371/journal.pgph.0003466 (PMC11288424; doi:10.1371/journal.pgph.0003466)
Supplement: S6 Table — (DOCX) [file pgph.0003466.s008.docx]

**Table S6: Prevalence of Ideal Cardiovascular Health Characteristics by Social Support Tertile**

| **ICH Characteristic** | **Tertile 1**  **% (CI)** | **Tertile 2**  **% (CI)** | **Tertile 3**  **% (CI)** | **P-value (difference between groups)** |
| --- | --- | --- | --- | --- |
| **Males and Females** |  |  |  |  |
| Normal BMI | 37.4 (29.8 – 45.1) | 38.9 (30.9 – 46.8) | 32.8 (25.4 – 40.2) | 0.491 |
| Non-Smoker | 78.8 (72.7 – 84.8) | 80.3 (74.3 – 86.3) | 83.2 (76.8 – 89.7) | 0.616 |
| Normal Glucose | 68.7 (60.2 – 77.2) | 63.9 (54.9 – 72.9) | 68.9 (58.8 – 78.9) | 0.647 |
| Normal Blood Pressure | 38.5 (31.3 – 45.7) | 33.1 (26.2 – 40.0) | 31.5 (22.4 – 40.7) | 0.273 |
| Adequate Physical Activity | 40.1 (30.9 – 49.0) | 39.3 (32.4 – 46.2) | 41.3 (33.7 – 48.9) | 0.932 |
| Healthy Diet | 15.9 (10.1 – 21.8) | 22.8 (16.4 – 29.3) | 19.7 (15.2 – 24.1) | 0.144 |
| Normal Cholesterol | 83.8 (77.6 – 90.1) | 79.9 (72.6 – 87.2) | 73.8 (67.3 – 80.3) | 0.112 |
|  |  |  |  |  |
| **Males** |  |  |  |  |
| Normal BMI | 53.3 (40.7 – 65.9) | 44.1 (33.3 – 55.1) | 39.4 (25.7 – 53.0) | 0.414 |
| Non-Smoker | 76.7 (64.7 – 88.8) | 60.9 (49.5 – 72.2) | 74.3 (64.9 – 83.8) | 0.111 |
| Normal Glucose | 72.3 (61.7 – 83.1) | 59.2 (45.9 – 72.6) | 67.1 (54.7 – 79.2) | 0.385 |
| Normal Blood Pressure | 37.9 (27.9 – 47.9) | 22.7 (8.4 – 37.1) | 26.6 (17.4 – 35.7) | 0.136 |
| Adequate Physical Activity | 58.9 (46.4 – 71.5) | 48.7 (33.4 – 64.1) | 48.6 (38.3 – 58.9) | 0.416 |
| Healthy Diet | 17.1 (7.9 – 26.1) | 18.8 (8.1 – 29.5) | 14.4 (8.6 – 20.2) | 0.759 |
| Normal Cholesterol | 80.0 (69.8 – 90.2) | 81.1 (71.1 – 91.2) | 77.7 (67.4 – 87.9) | 0.887 |
|  |  |  |  |  |
| **Females** |  |  |  |  |
| Normal BMI | 24.7 (16.7 – 32.7) | 34.5 (25.4 – 43.5) | 25.6 (19.8 – 31.5) | 0.298 |
| Non-Smoker | 80.4 (73.9 – 86.9) | 96.4 (93.4 – 99.3) | 92.8 (88.1 – 97.7) | <0.001 |
| Normal Glucose | 65.8 (55.5 – 76.0) | 67.8 (55.4 – 80.2) | 70.9 (60.6 – 81.2) | 0.784 |
| Normal Blood Pressure | 40.1(29.1 – 49.0) | 41.7 (32.9 – 50.5) | 40.1 (23.9 – 50.0) | 0.842 |
| Adequate Physical Activity | 24.7 (16.4 – 33.1) | 31.6 (20.8 – 42.3) | 33.4 (25.7 – 41.2) | 0.206 |
| Healthy Diet | 15.1 (7.6 – 22.4) | 26.2 (17.5 – 34.9) | 25.4 (18.9 – 31.8) | 0.136 |
| Normal Cholesterol | 86.8 (79.9 – 93.6) | 78.8 (69.6 – 88.0) | 69.6 (62.9 – 76.2) | 0.015 |

BMI = body mass index
