# Supplementary material for: Social support and ideal cardiovascular health in urban Jamaica: A cross-sectional study
Source: PLOS Glob Public Health. 2024 Jul 30;4(7):e0003466. doi: 10.1371/journal.pgph.0003466 (PMC11288424; doi:10.1371/journal.pgph.0003466)
Supplement: S7 Table — (DOCX) [file pgph.0003466.s009.docx]

**Table S7: Odds ratio for unit change in social support score for each Ideal Cardiovascular Health Characteristics in bivariate models.**

| ICH Characteristic | Males and Females  OR (95% CI) | Males  OR (95% CI) | Females  OR (95% CI) |
| --- | --- | --- | --- |
| Normal BMI | 1.0 (0.87 – 1.14) | 0.93 (0.76 – 1.13) | 1.04 (0.94 – 1.16) |
| Non-smoker | 0.91 (0.76 – 1.09) | 0.87 (0.69 – 1.06) | 1.94 (1.25 – 3.01) ** |
| Normal glucose | 0.97 (0.82 – 1.14) | 0.94 (0.77 – 1.14) | 1.06 (0.85 – 1.32) |
| Normal blood pressure | 0.97 (0.84 – 1.15) | 0.96 (0.77 – 1.21) | 1.07 (0.91 – 1.26) |
| Adequate physical activity | 1.0 (0.87 – 1.15) | 0.89 (0.75 – 1.06) | 1.10 (0.90 – 1.35) |
| Healthy diet | 1.05 (0.92 – 1.20) | 0.93 (0.75 – 1.16) | 1.26 (1.07 – 1.48) ** |
| Normal cholesterol | 0.90 (0.78 – 1.04) | 1.0 (0.82 – 1.23) | 0.74 (0.62 – 0.90) ** |

*p<0.05; **p<0.01; ***p<0.001
