# Supplementary material for: Social support and ideal cardiovascular health in urban Jamaica: A cross-sectional study
Source: PLOS Glob Public Health. 2024 Jul 30;4(7):e0003466. doi: 10.1371/journal.pgph.0003466 (PMC11288424; doi:10.1371/journal.pgph.0003466)
Supplement: S8 Table — (DOCX) [file pgph.0003466.s010.docx]

**Table S8: Odds ratio for unit change in social support score for each Ideal Cardiovascular Health Characteristics in multivariable models^1^.**

| ICH Characteristic | Males  OR (95% CI) | Females  OR (95% CI) |
| --- | --- | --- |
| Normal BMI | 0.96 (0.81 – 1.14) | 1.18 (1.02 – 1.34) * |
| Non-smoker | 0.80 (0.66 – 0.99) * | 1.73 (1.32 – 2.66) * |
| Normal glucose | 0.88 (0.69 – 1.11) | 1.16 (0.91 – 1.47) |
| Normal blood pressure | 0.98 (0.80 – 1.20) | 1.20 (0.97 – 1.50) |
| Adequate physical activity | 0.91 (0.76 – 1.08) | 1.07 (0.80 – 1.44) |
| Healthy diet | 0.90 (0.69 – 1.17) | 1.20 (1.00 – 1.44) * |
| Normal cholesterol | 1.04 (0.81 – 1.33) | 0.82 (0.69 – 0.98) * |

*p<0.05; **p<0.01; ***p<0.001

^1^Adjusted for age, education level, median property value and community poverty.
