# Supplementary material for: Social support and ideal cardiovascular health in urban Jamaica: A cross-sectional study
Source: PLOS Glob Public Health. 2024 Jul 30;4(7):e0003466. doi: 10.1371/journal.pgph.0003466 (PMC11288424; doi:10.1371/journal.pgph.0003466)
Supplement: S9 Table — (DOCX) [file pgph.0003466.s011.docx]

**Table S9: Survey weighted multivariable model without imputations^1^ showing the odds of having five or more ideal cardiovascular health characteristics (ICH-5) for males and females with their corresponding 95% CI and p-value.**

| **Variables** | **Females**  **Odds ratio (95% CI)** | **P-value** | **Males**  **Odds ratio (95% CI)** | **P-value** |
| --- | --- | --- | --- | --- |
| Social Support Score | 1.3 (1.1 – 1.6) | 0.004 | 0.7 (0.5 – 0.9) | 0.003 |
| Age | 0.9 (0.9-1.0) | 0.001 | 1.0 (0.9 – 1.0) | 0.005 |
| Education Category |  |  |  |  |
| Less than High School | Reference | Reference | Reference | Reference |
| High School | 0.4 (0.1 – 1.6) | 0.185 | 1.3 (0.6 – 3.1) | 0.486 |
| More Than High School | 0.7 (0.2 – 2.0) | 0.485 | 0.7 (0.2 – 2.4) | 0.555 |
| Land Value Category |  |  |  |  |
| Lower Category | Reference | Reference | Reference | Reference |
| Middle category | 1.3 (0.6 – 2.6) | 0.528 | 0.4 (0.1 – 1.3) | 0.130 |
| Upper Category | 2.5 (1.1 – 5.4) | 0.026 | 2.5 (0.8 – 7.5) | 0.096 |
| Community Poverty Category |  |  |  |  |
| Lower Category | Reference | Reference | Reference | Reference |
| Middle Category | 1.2 (0.5 – 2.8) | 0.705 | 5.2 (1.5 – 17.9) | 0.010 |
| Upper Category | 2.1 (0.7 – 6.0) | 0.168 | 1.9 (1.0 – 3.7) | 0.055 |

^1^Separate models created for 245 males and 495 females with ICH-5 as outcome, social support score and main exposure variable and adjusting for age, education, median land value and community poverty.
