# Supplementary material for: Social support and ideal cardiovascular health in urban Jamaica: A cross-sectional study
Source: PLOS Glob Public Health. 2024 Jul 30;4(7):e0003466. doi: 10.1371/journal.pgph.0003466 (PMC11288424; doi:10.1371/journal.pgph.0003466)
Supplement: S1 Dictionary — (DOCX) [file pgph.0003466.s012.docx]

**Code book for file “Data set for Social Support Paper_2023_10_05.dta"**

. codebook

--------------------------------------------------------------------------------------------------------------------------------

fasting_gluc_level 1 fasting_gluc_level_

--------------------------------------------------------------------------------------------------------------------------------

Type: Numeric (double)

Range: [1.4,23.3] Units: .1

Unique values: 97 Missing .: 325/1,130

Mean: 5.93789

Std. dev.: 2.30069

Percentiles: 10% 25% 50% 75% 90%

4.5 4.9 5.3 5.9 7.7

--------------------------------------------------------------------------------------------------------------------------------

tot_cholest_levels_new total cholesterol level

--------------------------------------------------------------------------------------------------------------------------------

Type: Numeric (float)

Range: [2.59,11.64] Units: .01

Unique values: 78 Missing .: 326/1,130

Mean: 4.39413

Std. dev.: 1.18677

Percentiles: 10% 25% 50% 75% 90%

2.9 3.5 4.3 5.1 5.9

--------------------------------------------------------------------------------------------------------------------------------

weight_kg weight in kilograms

--------------------------------------------------------------------------------------------------------------------------------

Type: Numeric (float)

Range: [39.5,245] Units: .1

Unique values: 487 Missing .: 306/1,130

Mean: 78.4968

Std. dev.: 22.8929

Percentiles: 10% 25% 50% 75% 90%

54.3 62.9 74.55 89.5 104.1

--------------------------------------------------------------------------------------------------------------------------------

height height in cm

--------------------------------------------------------------------------------------------------------------------------------

Type: Numeric (double)

Range: [112,193.6] Units: .1

Unique values: 326 Missing .: 312/1,130

Mean: 165.482

Std. dev.: 9.65126

Percentiles: 10% 25% 50% 75% 90%

154 159.4 165.4 172 177.5

--------------------------------------------------------------------------------------------------------------------------------

sex sex of participant (1 N12observedsexlp)

--------------------------------------------------------------------------------------------------------------------------------

Type: Numeric (byte)

Label: sex

Range: [0,1] Units: 1

Unique values: 2 Missing .: 281/1,130

Tabulation: Freq. Numeric Label

567 0 Female

282 1 Male

281 .

--------------------------------------------------------------------------------------------------------------------------------

age 1 age_last_bday_

--------------------------------------------------------------------------------------------------------------------------------

Type: Numeric (byte)

Range: [15,100] Units: 1

Unique values: 78 Missing .: 281/1,130

Mean: 47.6996

Std. dev.: 18.4666

Percentiles: 10% 25% 50% 75% 90%

22 32 48 62 73

--------------------------------------------------------------------------------------------------------------------------------

friendnumber number close friends and relatives you have

--------------------------------------------------------------------------------------------------------------------------------

Type: Numeric (byte)

Range: [0,50] Units: 1

Unique values: 25 Missing .: 282/1,130

Mean: 5.53538

Std. dev.: 5.14362

Percentiles: 10% 25% 50% 75% 90%

1 2 4 7 10

--------------------------------------------------------------------------------------------------------------------------------

friendloan number of friends/relatives to whom you can turn when you need to borrow somethi

--------------------------------------------------------------------------------------------------------------------------------

Type: Numeric (byte)

Range: [0,50] Units: 1

Unique values: 22 Missing .: 285/1,130

Mean: 4.65562

Std. dev.: 4.77339

Percentiles: 10% 25% 50% 75% 90%

1 2 3 6 10

--------------------------------------------------------------------------------------------------------------------------------

friendadvice number of friends/relatives who you can ask for advice or information

--------------------------------------------------------------------------------------------------------------------------------

Type: Numeric (byte)

Range: [0,55] Units: 1

Unique values: 23 Missing .: 284/1,130

Mean: 5.25768

Std. dev.: 5.13761

Percentiles: 10% 25% 50% 75% 90%

1 2 4 6 10

--------------------------------------------------------------------------------------------------------------------------------

socialintegration1 1 socialintegration1_

--------------------------------------------------------------------------------------------------------------------------------

Type: Numeric (byte)

Label: _socialintegration1

Range: [1,6] Units: 1

Unique values: 6 Missing .: 284/1,130

Tabulation: Freq. Numeric Label

96 1 Never

130 2 Less than once a month

133 3 About once a month

112 4 2 or 3 times a month

152 5 Once a week

223 6 More than once a week

284 .

--------------------------------------------------------------------------------------------------------------------------------

socialintegration2 1 socialintegration2_

--------------------------------------------------------------------------------------------------------------------------------

Type: Numeric (byte)

Label: _socialintegration2

Range: [1,6] Units: 1

Unique values: 6 Missing .: 282/1,130

Tabulation: Freq. Numeric Label

42 1 Never

51 2 Less than once a week

56 3 About once a week

147 4 2 or 3 times a week

72 5 Once a day

480 6 More than once a day

282 .

--------------------------------------------------------------------------------------------------------------------------------

lp_refusal (unlabeled)

--------------------------------------------------------------------------------------------------------------------------------

Type: Numeric (byte)

Range: [0,1] Units: 1

Unique values: 2 Missing .: 0/1,130

Tabulation: Freq. Value

849 0

281 1

--------------------------------------------------------------------------------------------------------------------------------

education_cat highest level of education attained

--------------------------------------------------------------------------------------------------------------------------------

Type: Numeric (byte)

Label: education_cat

Range: [1,3] Units: 1

Unique values: 3 Missing .: 291/1,130

Tabulation: Freq. Numeric Label

187 1 Less the High School

437 2 High School

215 3 More than High School

291 .

--------------------------------------------------------------------------------------------------------------------------------

smoke_current currently smokes any form of tobacco (cigarettes etc)?

--------------------------------------------------------------------------------------------------------------------------------

Type: Numeric (byte)

Label: true

Range: [0,1] Units: 1

Unique values: 2 Missing .: 281/1,130

Tabulation: Freq. Numeric Label

706 0 No

143 1 Yes

281 .

--------------------------------------------------------------------------------------------------------------------------------

non_smoker_ich never smoked cigarettes/tobacco or stopped 1 year or longer

--------------------------------------------------------------------------------------------------------------------------------

Type: Numeric (byte)

Label: true

Range: [0,1] Units: 1

Unique values: 2 Missing .: 281/1,130

Tabulation: Freq. Numeric Label

154 0 No

695 1 Yes

281 .

--------------------------------------------------------------------------------------------------------------------------------

phys_act_ich physical activity >=150 mod, >=75 vig, >=150 walk for adults or mean 60 min/day

--------------------------------------------------------------------------------------------------------------------------------

Type: Numeric (byte)

Label: true

Range: [0,1] Units: 1

Unique values: 2 Missing .: 298/1,130

Tabulation: Freq. Numeric Label

543 0 No

289 1 Yes

298 .

--------------------------------------------------------------------------------------------------------------------------------

low_salt_ich_ja low salt diet (no added salt at table & rarely or never eats processed foods)

--------------------------------------------------------------------------------------------------------------------------------

Type: Numeric (byte)

Label: true

Range: [0,1] Units: 1

Unique values: 2 Missing .: 286/1,130

Tabulation: Freq. Numeric Label

443 0 No

401 1 Yes

286 .

--------------------------------------------------------------------------------------------------------------------------------

healthy_diet_score4 healthy diet score using 4 variables (fruit fish sugar salt)

--------------------------------------------------------------------------------------------------------------------------------

Type: Numeric (byte)

Range: [0,4] Units: 1

Unique values: 5 Missing .: 293/1,130

Tabulation: Freq. Value

101 0

282 1

254 2

161 3

39 4

293 .

--------------------------------------------------------------------------------------------------------------------------------

healthy_diet_score5 healthy diet score using 5 variables (fruit fish sugar salt beans)

--------------------------------------------------------------------------------------------------------------------------------

Type: Numeric (byte)

Range: [0,5] Units: 1

Unique values: 6 Missing .: 293/1,130

Tabulation: Freq. Value

74 0

237 1

269 2

180 3

64 4

13 5

293 .

--------------------------------------------------------------------------------------------------------------------------------

healthy_diet_score5_fibre healthy diet score using 5 variables (fruit fish sugar salt fibre)

--------------------------------------------------------------------------------------------------------------------------------

Type: Numeric (byte)

Range: [0,5] Units: 1

Unique values: 6 Missing .: 293/1,130

Tabulation: Freq. Value

80 0

266 1

234 2

181 3

65 4

11 5

293 .

--------------------------------------------------------------------------------------------------------------------------------

healthy_diet_ich1 healthy diet for ICH >=3 of 4 variables (fruit fish sugar salt)

--------------------------------------------------------------------------------------------------------------------------------

Type: Numeric (byte)

Label: true

Range: [0,1] Units: 1

Unique values: 2 Missing .: 293/1,130

Tabulation: Freq. Numeric Label

637 0 No

200 1 Yes

293 .

--------------------------------------------------------------------------------------------------------------------------------

healthy_diet_ich2 healthy diet for ICH >=4 of 5 variables (fruit fish sugar salt fibre)

--------------------------------------------------------------------------------------------------------------------------------

Type: Numeric (byte)

Label: true

Range: [0,1] Units: 1

Unique values: 2 Missing .: 293/1,130

Tabulation: Freq. Numeric Label

761 0 No

76 1 Yes

293 .

--------------------------------------------------------------------------------------------------------------------------------

mn23sbp mean of 2nd & 3rd SBP readings

--------------------------------------------------------------------------------------------------------------------------------

Type: Numeric (float)

Range: [83.5,232] Units: .1

Unique values: 194 Missing .: 301/1,130

Mean: 130.893

Std. dev.: 22.4412

Percentiles: 10% 25% 50% 75% 90%

105 115 127 144.5 161

--------------------------------------------------------------------------------------------------------------------------------

mn23dbp mean of 2nd & 3rd DBP readings

--------------------------------------------------------------------------------------------------------------------------------

Type: Numeric (float)

Range: [51.5,154] Units: .1

Unique values: 134 Missing .: 301/1,130

Mean: 84.4264

Std. dev.: 13.5503

Percentiles: 10% 25% 50% 75% 90%

68.5 75 83 92.5 102

--------------------------------------------------------------------------------------------------------------------------------

normal_bp_ich Has normal blood pressure (SBP <120, DBP <80, no HTN self report)

--------------------------------------------------------------------------------------------------------------------------------

Type: Numeric (byte)

Label: true

Range: [0,1] Units: 1

Unique values: 2 Missing .: 19/1,130

Tabulation: Freq. Numeric Label

918 0 No

193 1 Yes

19 .

--------------------------------------------------------------------------------------------------------------------------------

bmi body mass index (weight_kg)/height_m)^2

--------------------------------------------------------------------------------------------------------------------------------

Type: Numeric (float)

Range: [15.022072,87.021873] Units: 1.000e-06

Unique values: 813 Missing .: 315/1,130

Mean: 28.7269

Std. dev.: 8.40214

Percentiles: 10% 25% 50% 75% 90%

19.8352 22.7174 27.2738 33.1315 38.6054

--------------------------------------------------------------------------------------------------------------------------------

bmi_normal_ich Has normal BMI

--------------------------------------------------------------------------------------------------------------------------------

Type: Numeric (byte)

Label: true

Range: [0,1] Units: 1

Unique values: 2 Missing .: 33/1,130

Tabulation: Freq. Numeric Label

835 0 No

262 1 Yes

33 .

--------------------------------------------------------------------------------------------------------------------------------

glucose_normal_ich normal glucose for ICH (<5.6 & no self reported DM)

--------------------------------------------------------------------------------------------------------------------------------

Type: Numeric (byte)

Label: true

Range: [0,1] Units: 1

Unique values: 2 Missing .: 322/1,130

Tabulation: Freq. Numeric Label

341 0 No

467 1 Yes

322 .

--------------------------------------------------------------------------------------------------------------------------------

chol_normal_ich chol <5.2 and no selfreported high cholesterol

--------------------------------------------------------------------------------------------------------------------------------

Type: Numeric (byte)

Label: true

Range: [0,1] Units: 1

Unique values: 2 Missing .: 326/1,130

Tabulation: Freq. Numeric Label

252 0 No

552 1 Yes

326 .

--------------------------------------------------------------------------------------------------------------------------------

socialintegration_mean social intergration index(mean of two variables)

--------------------------------------------------------------------------------------------------------------------------------

Type: Numeric (float)

Range: [1,6] Units: .1

Unique values: 11 Missing .: 284/1,130

Mean: 4.39066

Std. dev.: 1.30368

Percentiles: 10% 25% 50% 75% 90%

2.5 3.5 4.5 5.5 6

--------------------------------------------------------------------------------------------------------------------------------

ich_score_nomiss1 number of ICH variables among those will data on all 7 vars

--------------------------------------------------------------------------------------------------------------------------------

Type: Numeric (byte)

Range: [0,7] Units: 1

Unique values: 8 Missing .: 369/1,130

Tabulation: Freq. Value

3 0

60 1

161 2

242 3

158 4

110 5

26 6

1 7

369 .

--------------------------------------------------------------------------------------------------------------------------------

parishnumber (unlabeled)

--------------------------------------------------------------------------------------------------------------------------------

Type: Numeric (byte)

Range: [1,14] Units: 1

Unique values: 4 Missing .: 0/1,130

Tabulation: Freq. Value

395 1

480 2

48 3

207 14

--------------------------------------------------------------------------------------------------------------------------------

edid_grp group(parishnumber constituencynumber enumerationdistrict ed_id)

--------------------------------------------------------------------------------------------------------------------------------

Type: Numeric (int)

Range: [1,176] Units: 1

Unique values: 44 Missing .: 11/1,130

Mean: 46.9357

Std. dev.: 59.8865

Percentiles: 10% 25% 50% 75% 90%

5 12 23 34 172

--------------------------------------------------------------------------------------------------------------------------------

med_landvalue (mean) med_landvalue

--------------------------------------------------------------------------------------------------------------------------------

Type: Numeric (float)

Range: [.25,9] Units: .001

Unique values: 23 Missing .: 15/1,130

Mean: 1.60973

Std. dev.: 1.71351

Percentiles: 10% 25% 50% 75% 90%

.5 .6 1 1.7 2.7

--------------------------------------------------------------------------------------------------------------------------------

land_val_tert Land Value Tertiles (Area-specific)

--------------------------------------------------------------------------------------------------------------------------------

Type: Numeric (byte)

Label: tert_lab, but label does not exist

Range: [1,3] Units: 1

Unique values: 3 Missing .: 15/1,130

Tabulation: Freq. Value

605 1

306 2

204 3

15 .

--------------------------------------------------------------------------------------------------------------------------------

av_FGT0t Average Total Poverty (community)

--------------------------------------------------------------------------------------------------------------------------------

Type: Numeric (double)

Range: [.02368153,.3972694] Units: 1.000e-09

Unique values: 37 Missing .: 15/1,130

Mean: .218385

Std. dev.: .127753

Percentiles: 10% 25% 50% 75% 90%

.041318 .08975 .233645 .341484 .375108

--------------------------------------------------------------------------------------------------------------------------------

av_FGT0t_tert av_FGT0t Tertiles

--------------------------------------------------------------------------------------------------------------------------------

Type: Numeric (byte)

Label: tertile_lab

Range: [1,3] Units: 1

Unique values: 3 Missing .: 15/1,130

Tabulation: Freq. Numeric Label

400 1 Lower

220 2 Middle

495 3 Upper

15 .

--------------------------------------------------------------------------------------------------------------------------------

social_support_score1_pca Social Support Score from PCA

--------------------------------------------------------------------------------------------------------------------------------

Type: Numeric (float)

Range: [-1.773447,12.029449] Units: 1.000e-09

Unique values: 283 Missing .: 287/1,130

Mean: 3.5e-09

Std. dev.: 1.55804

Percentiles: 10% 25% 50% 75% 90%

-1.31504 -.979459 -.393157 .296987 1.67728

--------------------------------------------------------------------------------------------------------------------------------

no_DWs Total number of dwellings in ED (country)

--------------------------------------------------------------------------------------------------------------------------------

Type: Numeric (int)

Range: [65,518] Units: 1

Unique values: 38 Missing .: 3/1,130

Mean: 164.546

Std. dev.: 84.495

Percentiles: 10% 25% 50% 75% 90%

92 116 150 174 255

--------------------------------------------------------------------------------------------------------------------------------

fpc_urban_ed Number of urban EDs in parish

--------------------------------------------------------------------------------------------------------------------------------

Type: Numeric (int)

Range: [68,904] Units: 1

Unique values: 4 Missing .: 0/1,130

Tabulation: Freq. Value

48 68

395 234

207 769

480 904

--------------------------------------------------------------------------------------------------------------------------------

urb_popsize_par123_14_agec5sex Population size (5yr age-sex-parish)

--------------------------------------------------------------------------------------------------------------------------------

Type: Numeric (float)

Range: [257.38058,26272.346] Units: .00001

Unique values: 98 Missing .: 281/1,130

Mean: 9539.44

Std. dev.: 7790.37

Percentiles: 10% 25% 50% 75% 90%

1127.57 3065.91 7248.33 16847.9 21740.5

--------------------------------------------------------------------------------------------------------------------------------

sampwt_adjnr_prob_selection (unlabeled)

--------------------------------------------------------------------------------------------------------------------------------

Type: Numeric (float)

Range: [65.20799,3027.0059] Units: .00001

Unique values: 52 Missing .: 281/1,130

Mean: 567.376

Std. dev.: 549.369

Percentiles: 10% 25% 50% 75% 90%

107.64 159.003 431.987 784.196 1094.14

--------------------------------------------------------------------------------------------------------------------------------

postrata group(parishnumber male age_cat5)

--------------------------------------------------------------------------------------------------------------------------------

Type: Numeric (byte)

Range: [1,99] Units: 1

Unique values: 99 Missing .: 281/1,130

Mean: 38.0707

Std. dev.: 26.4679

Percentiles: 10% 25% 50% 75% 90%

7 14 34 51 81

--------------------------------------------------------------------------------------------------------------------------------

hh_unique (unlabeled)

--------------------------------------------------------------------------------------------------------------------------------

Type: Numeric (int)

Range: [1,1130] Units: 1

Unique values: 1,130 Missing .: 0/1,130

Mean: 565.5

Std. dev.: 326.347

Percentiles: 10% 25% 50% 75% 90%

113.5 283 565.5 848 1017.5

--------------------------------------------------------------------------------------------------------------------------------

strata_ed (unlabeled)

--------------------------------------------------------------------------------------------------------------------------------

Type: Numeric (int)

Range: [1,176] Units: 1

Unique values: 44 Missing .: 11/1,130

Mean: 46.9357

Std. dev.: 59.8865

Percentiles: 10% 25% 50% 75% 90%

5 12 23 34 172

--------------------------------------------------------------------------------------------------------------------------------

include2 Included in Social Support Analysis

--------------------------------------------------------------------------------------------------------------------------------

Type: Numeric (byte)

Label: true

Range: [0,1] Units: 1

Unique values: 2 Missing .: 0/1,130

Tabulation: Freq. Numeric Label

289 0 No

841 1 Yes

--------------------------------------------------------------------------------------------------------------------------------

ich_score_new_mi (unlabeled)

--------------------------------------------------------------------------------------------------------------------------------

Type: Numeric (byte)

Range: [0,7] Units: 1

Unique values: 8 Missing .: 289/1,130

Tabulation: Freq. Value

4 0

83 1

186 2

260 3

168 4

113 5

26 6

1 7

289 .

--------------------------------------------------------------------------------------------------------------------------------

ich_cat7_mi (unlabeled)

--------------------------------------------------------------------------------------------------------------------------------

Type: Numeric (byte)

Range: [0,1] Units: 1

Unique values: 2 Missing .: 289/1,130

Tabulation: Freq. Value

840 0

1 1

289 .

--------------------------------------------------------------------------------------------------------------------------------

ich_cat5_mi (unlabeled)

--------------------------------------------------------------------------------------------------------------------------------

Type: Numeric (byte)

Range: [0,1] Units: 1

Unique values: 2 Missing .: 289/1,130

Tabulation: Freq. Value

701 0

140 1

289 .

**LABEL LIST**

education_cat:

1 Less the High School

2 High School

3 More than High School

sex:

0 Female

1 Male

true:

0 No

1 Yes

_socialintegration1:

1 Never

2 Less than once a month

3 About once a month

4 2 or 3 times a month

5 Once a week

6 More than once a week

99 No response

88 Don't know

_socialintegration2:

1 Never

2 Less than once a week

3 About once a week

4 2 or 3 times a week

5 Once a day

6 More than once a day

99 No response

tertile_lab:

1 Lower

2 Middle

3 Upper
