# Supplementary material for: Social support and ideal cardiovascular health in urban Jamaica: A cross-sectional study
Source: PLOS Glob Public Health. 2024 Jul 30;4(7):e0003466. doi: 10.1371/journal.pgph.0003466 (PMC11288424; doi:10.1371/journal.pgph.0003466)
Supplement: S1 Questionnaire — (PDF) [file pgph.0003466.s015.pdf]

Cardiovascular Health in Urban Jamaica: Psychosocial Stress, Social Networks and Social Support

Investigators: Trevor S Ferguson, Marshall K Tulloch-Reid, Novie O Younger-Coleman, Ishtar Govia, Joette A McKenzie, Shelly R McFarlane, Damian K Francis, Rainford J Wilks, David R. Williams

Consent Form

Purpose of Study: Researchers from the Epidemiology Research Unit of the Caribbean Institute for Health Research at the University of the West Indies in Jamaica and the Harvard School of Public Health in the United States are carrying out a community-based research on persons 15 and older in urban Jamaica. The purpose of this study is to find how the health of people's heart and blood vessels is related to factors such as the income level of the community they live in, their stress level, and the amount of support they receive from family and friends, or from participation in church or other religious communities. Findings from this study will help in identifying things that the government or other organizations could do to improve people's health.

Procedures: You are being asked to be a part of this study, because you were a participant in the Jamaica Health and Lifestyle Survey which was conducted between mid-2016 and early 2017 or because you live in one of the communities in which the study was done. If you agree to participate in this study, we will use information collected in the previous study along with additional information to be collected now in our research.

Firstly, we will ask you to answer a set of questions to identify stressful factors that you may have experienced, estimate your stress level and the level of support you receive from family, friends or your religious community. Questions will include major events in your life, experiences on the job, feelings about unfair treatment and feelings about your relationships. We will also ask about receiving or giving help to family, friends or your religious community. You have the right to refuse to answer any question, which makes you feel uncomfortable.

Secondly, we will measure your blood pressure, height, weight, hip, and waist size. We will also ask you to give a finger prick blood sample to measure blood sugar and cholesterol levels.

Lastly, we will collect information about your community from government or other sources to help us place communities in income categories. This information will be about your community in general and not specific to you. No personal information will be collected.

The interview will be conducted in your home or at another convenient location. The entire interview (questionnaire and measurements) should take about forty-five minutes. The finger stick sample will require another five to 10 minutes. We would like to do the finger stick first thing in the morning, before you have eaten, so we will arrange the interview in the early morning or come back another morning to do it. Overall the study will be completed during one or two visits.

Your participation is entirely up to you. You may choose not to participate in the any part of the study or to stop participating if you do not wish to continue, even after you have started.

Benefits: There are no definite benefits to you for participating in this study but the research is likely to be beneficial to society, if the findings lead to the introduction of new policies or procedures to improve the nation's health. The tests performed may have some indirect benefits, in that, if we find anything abnormal with you during the study we will refer you to the appropriate health department for further care. Such referral will be made to the public health service, so there would be no associated user fee. If you prefer to be referred to another health care facility, this will be at you own expense.

Risks: There are minimal risks associated with this study. You may experience brief discomfort from the finger stick and some questions may make you feel a bit uncomfortable or cause you to remember unpleasant events. If you feel uncomfortable about any of the questions, the interviewer can skip those questions and go on to the next section. For the finger stick test a new sterile (clean) lancing device will be used.

Confidentiality: All information you provide will be treated with strict confidentiality and will be available only to the researchers on this project. All files will be kept in locked filing cabinets or on password protected computers. Personal identifying information, such as your name and address, will be kept separately from the other information you have given. The findings from the study will be put together in a report and published in medical journals, but we will report only information about the study as a whole, or in broad subgroups, so that it will not be possible to identify your information.

Contact Numbers: This study has been approved by the Ethics committee of the University of the West Indies (UWI). If you have any questions about the research or your participation, contact Dr Trevor Ferguson of the Caribbean Institute for Health Research at (876) 9272471, or by email at: trevor.ferguson02@uwimona.edu.jm

For independent advice as a research participant, you may contact the Chair of the UWI Ethics Committee and Dean of the Faculty of Medical Sciences, UWI, Dr Tomlin Paul at (876) 927-2556 or via email at ethics.committee@uwimona.edu.jm.

DECLARATION

I have read, or had this form read to me. I was given the opportunity to ask questions which were answered to my satisfaction. I voluntarily give permission to participate in this research project and indicate this by signing on the designated space, below. I am entitled to be given a copy of this form if I so desire.

Participant

QUESTIONNAIRE ID NO. |___|___|___|___|___|___|___|___|___|___|___|___|___|

Reg Par Con ED Dw H Re

Name : ______________________________ DOB |__||__||__|__||__|__|__|__|
D D M M Y Y Y Y

Address: ______________________________

Signature: ______________________________ Date: ___/_____/_____
DD MM YY

Witness

Name: ______________________________

Address: ______________________________

Signature: ______________________________ Date: ___/_____/_____
DD MM YY

Phone number: ____________________ (main) _________________ (secondary)

Name of family member or other person we may contact to find you: ________________________

Contact person's phone number ____________________ Relation _______________

Cardiovascular Health in Urban Jamaica: Psychosocial Stress, Social Networks and Social Support

Investigators: Trevor S Ferguson, Marshall K Tulloch-Reid, Novie O Younger-Coleman, Ishtar Govia, Joette A McKenzie, Shelly R McFarlane, Damian K Francis, Rainford J Wilks, David R. Williams

Consent Form

Purpose of Study: Researchers from the Epidemiology Research Unit of the Caribbean Institute for Health Research at the University of the West Indies in Jamaica and the Harvard School of Public Health in the United States are carrying out a community-based research on persons 15 and older in urban Jamaica. The purpose of this study is to find how the health of people's heart and blood vessels is related to factors such as the income level of the community they live in, their stress level, and the amount of support they receive from family and friends, or from participation in church or other religious communities. Findings from this study will help in identifying things that the government or other organizations could do to improve people's health.

Procedures: You are being asked to be a part of this study, because you were a participant in the Jamaica Health and Lifestyle Survey which was conducted between mid-2016 and early 2017 or because you live in one of the communities in which the study was done. If you agree to participate in this study, we will use information collected in the previous study along with additional information to be collected now in our research.

Firstly, we will ask you to answer a set of questions to identify stressful factors that you may have experienced, estimate your stress level and the level of support you receive from family, friends or your religious community. Questions will include major events in your life, experiences on the job, feelings about unfair treatment and feelings about your relationships. We will also ask about receiving or giving help to family, friends or your religious community. You have the right to refuse to answer any question, which makes you feel uncomfortable.

Secondly, we will re-measure your blood pressure, height, weight, hip, and waist size. We will also ask you to give a finger prick blood sample to measure blood sugar and cholesterol levels.

Lastly, we will collect information about your community from government or other sources to help us place communities in income categories. This information will be about your community in general and not specific to you. No personal information will be collected.

The interview will be conducted in your home or at another convenient location. The entire interview (questionnaire and measurements) should take about forty-five minutes. The finger stick sample will require another five to 10 minutes. We would like to do the finger stick first thing in the morning, before you have eaten, so we will arrange the interview in the early morning or come back another morning to do it. Overall the study will be completed during one or two visits.

Your participation is entirely up to you. You may choose not to participate in the any part of the study or to stop participating if you do not wish to continue, even after you have started.

Benefits: There are no definite benefits to you for participating in this study but the research is likely to be beneficial to society, if the findings lead to the introduction of new policies or procedures to improve the nation's health. The tests performed may have some indirect benefits, in that, if we find anything abnormal with you during the study we will refer you to the appropriate health department for further care. Such referral will be made to the public health service, so there would be no associated user fee. If you prefer to be referred to another health care facility, this will be at you own expense.

Risks: There are minimal risks associated with this study. You may experience brief discomfort from the finger stick and some questions may make you feel a bit uncomfortable or cause you to remember unpleasant events. If you feel uncomfortable about any of the questions, the interviewer can skip those

questions and go on to the next section. For the finger stick test a new sterile (clean) lancing device will be used.

Confidentiality: All information you provide will be treated with strict confidentiality and will be available only to the researchers on this project. All files will be kept in locked filing cabinets or on password protected computers. Personal identifying information, such as your name and address, will be kept separately from the other information you have given. The findings from the study will be put together in a report and published in medical journals, but we will report only information about the study as a whole, or in broad subgroups, so that it will not be possible to identify your information.

Contact Numbers: This study has been approved by the Ethics committee of the University of the West Indies (UWI). If you have any questions about the research or your participation, contact Dr Trevor Ferguson of the Caribbean Institute for Health Research at (876) 9272471, or by email at: trevor.ferguson02@uwimona.edu.jm

For independent advice as a research participant, you may contact the Chair of the UWI Ethics Committee and Dean of the Faculty of Medical Sciences, U.W.I, Dr Tomlin Paul at (876) 927-2556 or via email at ethics.committee@uwimona.edu.jm.

DECLARATION

I have read, or had this form read to me. I was given the opportunity to ask questions which were answered to my satisfaction. I voluntarily give permission to participate in this research project and indicate this by signing on the designated space, below. I am entitled to be given a copy of this form if I so desire.

Participant

QUESTIONNAIRE ID NO. |___|___|___|___|___|___|___|___|___|___|___|___|___|
Reg Par Con ED Dw H Re

Name : ______________________________ DOB |__||__||__|__||__|__|__|__|
D D M M Y Y Y Y

Address: ______________________________

Signature: ______________________________ Date: ____/______/______
DD MM YY

Witness

Name: ______________________________

Address: ______________________________

Signature: ______________________________ Date: ____/______/______
DD MM YY

Phone number: ____________________ (main) ____________________ (secondary)

Cardiovascular Health in Urban Jamaica: Psychosocial Stress, Social Networks and Social Support

ID NO. |___|___|___|___|___|___|___|___|___|___|___|___|___| QUESTIONNAIRE ID NO. [__]__] __] __]
Reg Par Con ED Dw H Re

PARISH ________________________ What is the name of the area you live in:_______________

REGION NO.	|____|	DWELLING NO.	|____|____|____|
PARISH NO.<br>CONSTITUENCY NO.	|____|____| |____|____|	HOUSEHOLD NO.	|____|
ENUMERATION DISTRICT NO.	|___|___|___|	RESPONDENT NO.	|____|

**Interview Visit**	**Visit 1**	**Visit 2**	**Visit 3**	**Final Visit**
Date (dd/mmm/yy)				
Status of Visit				
Interviewer's Initials				
Interviewer's ID Number				
Date and Time of next appt.				

┼ **Interview Status codes**	╬ **Refusal Status Complete refusals**	╬ **Refusal Status Partial Refusals**
1. Completed household interviews : assessment of eligibility<br>2. No individual available for household interview - **REVISIT**<br>3. Household interviews completed – selected respondent not at home or available - REVISIT<br>4. Partially completed questionnaire – REVISIT<br>5. Partially completed questionnaire – anthropometry and biomedical measures outstanding<br>6. Partially completed questionnaire- anthropometry outstanding<br>7. Partially completed questionnaire- biomedical measure incomplete<br>8. Refusal – individual<br>9. Completed household interview - selected respondent –completed questionnaire	1. Study not relevant to me<br>2. No time, too busy<br>3. Invasion of privacy<br>4. Too personal<br>5. Didn't believe information was confidential<br>6. Other (specify)	1. Questionnaire incomplete<br>2. Anthropometry<br>3. Blood pressure<br>4. Biomedical measures refused

START TIME OF INTERVIEW- HOUR [__] MINUTES [__] [__] AM/PM

END TIME OF INTERVIEW - HOUR [__] MINUTES [__] [__] AM/PM

Please circle the correct time of day

From Jamaica Health and Lifestyle Survey
Used with permission

ID NO. |___|___|___|___|___|___|___|___|___|___|___|___|___| QUESTIONNAIRE ID NO. [__]__] __] __]
Reg Par Con ED Dw H Re

TOTAL TIME OF INTERVIEW – HOURS [__] MINUTES [__] [__]

KISH TABLE

How many eligible persons live in this household? _______ Number

PLEASE RECORD THE NAMES AND OTHER PARTICULARS OF ALL ELIGIBLE PERSONS WHO LIVE IN THIS HOUSEHOLD. THIS SHOULD INCLUDE ALL WHO USUALLY EAT AND SLEEP HERE. LIST ALL MALES FIRST, START WITH THE OLDEST MALE MEMBER, THEN THE NEXT OLDEST, THEN All FEMALES

SCHEDULE OF ALL PERSONS LIVING IN HOUSEHOLD * Sex codes **1**- Male **2**-Female

Line NO.	Name<br>*Please give me all the names of all persons who usually live in your household*	*<br>*male or female*	AGE<br>*How old is this person*
01			
02			
03			
04			
05			
06			
07			
08			
09			
10			
11			
12			

IF THERE IS ONE OR MORE ELIGIBLE RESPONDENTS, SELECT THE ONE TO BE INTERVIEWED, BASED ON THE INSTRUCTIONS GIVEN AND USING THE RANDOM TABLE SHOWN BELOW.
THEN COMPLETE THE INDIVIDUAL QUESTIONNAIRE FOR THE SELECTED RESPONDENT
IF AN ELIGIBLE RESPONDENT HAS REFUSED, COMPLETE THE TITLE PAGE AND MOVE ON TO THE NEXT HOUSEHOLD

RANDOM SELECTION OF RESPONDENT
Questionnaire Number _______

Number of eligible persons _______

RANDOM SELECTION TABLE

**Last digit on questionnaire number**	**Number Of Eligible Persons In Household** 1	2	3	4	5	6	7	8	9	10
0	1	2	3	1	2	5	2	8	7	10
1	1	1	1	2	3	6	3	1	8	1
2	1	2	2	3	4	1	4	2	9	2
3	1	1	3	4	5	2	5	3	1	3
4	1	2	1	1	1	3	6	4	2	4
5	1	1	2	2	2	4	7	5	3	5
6	1	2	3	3	3	5	1	6	4	6
7	1	1	1	4	4	6	2	7	5	7
8	1	2	2	1	5	1	3	8	6	8
9	1	1	3	2	1	2	4	1	7	9

SEQUENCE NUMBER OF PERSONS SELECTED FOR INTERVIEW: _______

ID NO. |___|___|___|___|___|___|___|___|___|___|___|___|___| QUESTIONNAIRE ID NO. [__]__] __] __]
Reg Par Con ED Dw H Re

FORM 1: PULSE AND BLOOD PRESSURE MEASUREMENT

QUESTIONNAIRE ID NO.		I.D. OF INTERVIEWER |___|___|
DATE OF EXAM _____/_____/_____<br>DD MM YY		

Now I will explain the procedure for measuring your pulse and blood pressure. It is important that you remain relaxed and seated for the measurement which will take about 15 minutes. Please do not cross your feet or legs during the measurements. I will wrap the blood pressure cuff around your arm, take your pulse and then inflate the cuff. You will feel a sensation of pressure on your arm when the cuff is inflated. I will be inflating the cuff a maximum of 5 times. While I am measuring your blood pressure, it is best if we do not talk. If you have any questions, I will be happy to answer them for you before or after the measurement is taken. I will tell you the results of the measurements afterward.

1. Have you had any food, alcohol, coffee or cigarettes within the last 30 minutes?	Food: 0 [ ] N 1 [ ] Y<br>Alcohol: 0 [ ] N 1 [ ] Y<br>Coffee: 0 [ ] N 1 [ ] Y<br>Cigarettes: 0 [ ] N 1 [ ] Y
2. Arm circumference:	[____|____|____| . |____|
3. Cuff size selected:	0 [ ] Small adult 9 (18 – 25 cm)<br>1 [ ] Adult (25 – 35 cm)<br>2 [ ] Large (33 – 47 cm)<br>3 [ ] Thigh (>47 cm)
4. Arm selected:	0 [ ] Right 1 [ ] Left<br>______________________ Reason
5. First blood pressure measurement:<br>0 [ ] BP refused – Reason : ______________<br>1 [ ] BP not done – Reason: _____________	|____|____|____| / |____|____|____| SBP DBP
5. Pulse rate:	|____|____|____|
6. Second blood pressure measurement:	|____|____|____| / |____|____|____| SBP DBP
6. Pulse rate for 30 seconds:	|____|____|____|
7. Third blood pressure measurement:	|____|____|____| / |____|____|____| SBP DBP
7. Pulse rate for 30 seconds:	|____|____|____|

FORM 2: BIOMEDICAL MEASURES

1. What time and date did you last eat? TIME: |____|____| : |____|____| 0 [ ] AM
1 [ ] PM
DATE: _____/_____/_____
DD MMM YY

2. What time and date did you last have something other than water to drink? TIME: |____|____| : |____|____| 0 [ ] AM
1 [ ] PM
DATE: _____/_____/_____
DD MMM YY

3. What time and date did you last smoke? TIME: |____|____| : |____|____| 0 [ ] AM
1 [ ] PM
DATE: _____/_____/_____
DD MMM YY

Time of fasting sample: |____|____| : |____|____| 0 [ ] AM
1 [ ] PM

Fasting glucose level: |____|____|. |____| mmol/L

Total Cholesterol levels: |____|____|. |____|____| | mmol/L

COMMENTS: _

From Jamaica Health and Lifestyle Survey
Used with permission

FORM 3: BODY MEASUREMENTS AND PHYSICAL ACTIVITY

Now I am going to measure your height, weight, and waist and hip measurements. I will explain each one as we do it

WEIGHT<br><br>RECORD SCALE IDENTIFICATION NUMBER	|____|____|____| . |____| kg<br><br>|____|____|
HEIGHT	|____|____|____| . |____| cm
WAIST CIRCUMFERENCE<br><br><br>What clothing was the measurement taken over?	1. |____|____|____| . |____| cm<br>2. |____|____|____| . |____| cm<br>3. |____|____|____| . |____| cm<br><br>0 [ ] No clothing: skin<br>1 [ ] Shirt or dress<br>2 [ ] Trousers only<br>3 [ ] Shirt & trousers
BUTTOCKS (HIP) CIRCUMFERENCE<br><br><br>What clothing was the measurement taken over?	1. |____|____|____| . |____| cm<br>2. |____|____|____| . |____| cm<br>3. |____|____|____| . |____| cm<br><br>0 [ ] No clothing: skin<br>1 [ ] Shirt or dress<br>2 [ ] Trousers only<br>3 [ ] Shirt & trousers
Thickness of upper body covering:	0 [ ] None 1 [ ] Thin<br>2 [ ] Thick
Thickness of lower body covering:	0 [ ] None 1 [ ] Thin<br>2 [ ] Thick

From Jamaica Health and Lifestyle Survey
Used with permission

ID NO. |___|___|___|___|___|___|___|___|___|___|___|___|___| QUESTIONNAIRE ID NO. [__]__] __] __]
Reg Par Con ED Dw H Re

DEMOGRAPHIC INFORMATION (JHLS-III)

1.2 OBSERVED SEX: 1. Male 2. Female

**1.3** BIRTHDATE: _____/____/_____<br>DD MMM YY	**1. 4** AGE (At last birthday): _________Years

1.5 What is your union status? **(Circle only one)** ***PROMPT IF NECESSARY***| |__|__|

0. None (Single) 1. Married
2. Common law 3. Widowed
4. Divorced 5. Separated
6. Visiting
88. Don't know **99. No response**

1.6 What is the highest level or grade you have reached in school? **(Circle only one)** |__|__|
PROMPT IF NECESSARY

0. No schooling (Go to Q1.9) 1. Basic School
2. Primary 3. All Age/Junior High
4. Secondary/High School 5. Technical/Vocational school
6. College/tertiary 8. Other (Specify) _________________ |__|__|
88. Don't know **99. No response**

1.7 How many years did you spend at**? (Probe, confirm total number of years spent in each institution)**

**Institution**	**# years**	**Don't Know/remember**	**No response**
Basic School			
Primary School			
Secondary School			
Post –secondary			
Other, specify			

1.8 What is the highest examination that you passed? |__|__|

0. None 1. GSAT/Common Entrance/11+
2. Grade 9 Achievement 3. School Certificate, CXC Basic
4. O-Level/CXC Gen, CSEC 5. A-Levels /CAPE
6. College diplomas, Certificates 8. University degrees, Professional qualifications
9. Other (Specify) ______________ |__|__|
88. Don't know **99. No response**

From Jamaica Health and Lifestyle Survey
Used with permission

1.9 What is your employment status? **PROMPT IF NECESSARY; NB Can choose more than one**

1. Full-time (30 or more hours/week)
2. Part-time (29 or fewer hours/week)
3. Seasonally employed
4. Unemployed and looking **(Go to Q 3.1)**
5. Unemployed and not looking (**Go to Q 3.1)**
6. Student **(Go to Q 3.1)**
7. Retired **(Go to Q 1.9b)**
8. Other, specify _________________

88. Don't know
99. No response

1.9b If retired, what was your previous occupation? **(Go to Q 3.1)**

____

1.10 What is your primary occupation, that is the job which you spend most time doing? (***NB job includes any activity that you do to make money; i.e. your primary source of income.)***

____ |__|__|

1.11 Are you self-employed in this occupation? (i.e. job given in 1.10 above) |__|__|

0. No
1. Yes

88. Don't know
99. No response

1.12 Do you have another occupation? |__|__|

0. No **(Go to Q 3.1)**
1. Yes

88. **Don't know (Go to Q 3.1)**
99. **No response (Go to Q 3.1)**

1.13 What is your secondary occupation?

____ |__|__|

1.14 Are you self-employed in this occupation? |__|__|

0. No
1. Yes

88**. Don't know**
99. **No response**

1.15 What is your employment status in this occupation? PROMPT IF NECESSARY |__|__|

1. Full-time (30 or more hours/week)
2. Part-time (29 or fewer hours/week)
3. Seasonally employed
4. Other _______________ (specify)|__|__|

88. **Don't know**
99. **No response**

MEDICAL HISTORY (JHLS-III)

3.1. Has a health provider/doctor ever told that you have any of the following? Please respond to all items

(a) Heart Disease 0. No 1. Yes **88. Don't know** **99. No Response** [__]

(b) Diabetes Mellitus (sugar) 0. No 1. Yes **88. Don't know** **99. No Response** [__]

(d) High Blood Pressure 0. No 1. Yes **88. Don't know** **99. No Response** [__]

(e) High Cholesterol 0. No 1. Yes **88. Don't know** **99. No Response** [__]

(f) Stroke 0. No 1. Yes **88. Don't know** **99. No Response** [__]

(g) Heart Attack 0. No 1. Yes **88. Don't know** **99. No Response** [__]

(h) Obesity/Overweight 0. No 1. Yes **88. Don't know** **99. No Response** [__]

SMOKING HISTORY

10.12 Do you currently smoke any form of tobacco (cigarettes, cigars, beady etc.)? |__|__|
0. Never smoked **(Go to Q 12.1)** 1. No, Former, smoker
2. Yes, not every day 3. Yes, daily
88. Don't know (Go to Q 12.1) **99. No response (Go to Q 12.1)**

10.13 About how old were you when you first started smoking tobacco products (cigarettes, beady etc)? |__|__|
Age in years ______
88. Don't know/don't remember **99.No response**

10.14 Have you tried to stop smoking tobacco products?
0. No **(Go to Q 10.16)** 1. Yes, no longer smoke
2. Yes, still smoke **(Go to Q 10.16)**
88**. Don't know (Go to Q 10.16)** 99. **No response (Go to Q 10.16)**

10.15 When did you stop smoking tobacco products? (Number of months/years) **PROMPT** |__|__|
1. Less than 1 month ago 2. 1 – 5 months ago
3. 6 – 11 months ago 4. 1 – 3 years ago
5. 4 – 5 years ago 6. More than 5 years ago
88. Don't know **99. No response**

10.16 On average how many of the following tobacco products do you smoke and how frequently? (Write the frequency in the table provided)

**Product**	**0. Never Smokes**	**1. Daily**	**2. Weekly**	**3. Monthly**	**88. Don't Know**	**99. No response**
Manufactured Cigarettes						
Hand-rolled cigarettes/Beady						
Pipes						
Cigars,						
Shisha/Hooka						
E-cigarettes						
Other, specify_______						

10.17 Have you smoked at least 100 cigarettes in your life? |__|__|
0. No
1. Yes
88. Don't know
99. No response

PHYSICAL ACTIVITY LEVELS

12.1 When you consider your body weight, do you consider yourself to be - **PROMPT** |__|__|
1. The right weight
2. A little overweight
3. A lot overweight
4. Under weight
88. Don't know
99. No response

12.2. What is your main leisure time activity? **(Circle only one) PROMPT** |__|__|
1. Read, watch television and do things that do not require physical activity.
2. Walk, ride a bicycle or other physical activity for at least 4 hours a week. *(E.g. walking, fishing and hunting, light garden work, etc.)*
3. Physical activities to maintain fitness, *(e.g. running, gymnastics, dancing, swimming, ball games or doing heavy garden work or its equivalent).*
4. Regular training, several days a week, for competitions (*e.g. running, ball games or other physically heavy sports)*
88. Don't know
99. No response

12.3 How many times a week are you engaged in the activities you mentioned? **INTERVIEWER WILL REMIND PARTICIPANT OF LEISURE TIME ACTIVITY MENTIONED** |__|__|
0. Never
1. Less than once per week
2. 1 – 3 times a week
3. 4 – 6 times a week, Daily
88. Don't know
99. No response

12.4 How many minutes a day do you spend walking, cycling or in any other physical activity on your way to work? (Include both the time spent going to and coming from work***) PROMPT***

|__|__|

0. I don't work or get physical activity on the way to work
1. Less than 15 minutes a day
2. 15 – 29 minutes a day
3. 30 – 44 minutes a day
4. 45 – 59 minutes a day
5. One hour or more a day

88. Don't know
99. No response

Think about all the Vigorous activities that you did in the last 7 days. Vigorous activities refer to activities that take extra physical effort and make you breathe much harder than normal. Think only about those physical activities that you did for at least 10 minutes at a time.

12.8 During the last 7 days, on how many days did you do vigorous physical activities like heavy lifting, digging, running, basketball, football, tennis, or fast bicycling***?*** |__|__|

(a) 0. [___] No vigorous physical activities **(Go to Q 12.9)** 1. ___ **days per week**

(b) How much time did you usually spend doing vigorous physical activities on one of those days? |__|__|

___Hours per day **___ ___ ___ minutes per day**

88. Don't know/Not sure **99. No response**

Think about all the moderate activities that you did in the last 7 days. Moderate activities refer to activities that take moderate physical effort and make you breathe somewhat harder than normal. Think only about those physical activities that you did for at least 10 minutes at a time.

12.9 During the *last 7 days*, on how many days did you do *moderate* physical activities like carrying light loads, bicycling at a regular pace, doubles tennis? Do not include walking. |__|__|

(a) 0. No moderate physical activities **(Go to Q 12.10)** 1. ___ **Days per week**

(b) How much time did you usually spend doing moderate physical activities on one of those days?

___ Hours per day **___ ___ ___ minutes per day**

88. Don't know/Not sure **99. No response**

Think about the time you spent walking in the last 7 days. This includes at work and at home, walking to travel from place to place, and any other walking that you might do solely for recreation, sport, exercise, or leisure.

12.10 During the last 7 days, on how many days did you walk for at least 10 minutes at a time? |__|__|

(a) 0. No walking ___ **days per week**
88. Don't know/Not sure **99. No response**

(b)How much time did you usually spend **walking** on one of those days? |__|__|
___ **Hours per day** ___ ___ ___ **minutes per day**
88. Don't know/Not sure **99. No response**

The last question is about the time you spent sitting on weekdays during the last 7 days. Include time spent at work, at home, while doing course work and during leisure time. This may include time spent sitting at a desk, visiting friends, reading, or sitting or lying down to watch television.

12.11 During the last 7 days, how much time did you spend sitting on a week day?
___ **Hours per day** ___ ___ ___ **minutes per day**

88. Don't know/Not sure **99. No response**

DIETARY PRACTICES

These next questions are about the fruits and vegetables you ate or drank during the past 30 days. Please think about all forms of fruits and vegetables including cooked or raw, fresh, frozen or canned. Please think about all meals, snacks, and food consumed at home and away from home.

13.6 During the past month, how many times per day, week or month did you drink 100% PURE fruit juices? Do not include fruit-flavoured drinks with added sugar or fruit juice you made at home and added sugar to. Only include 100% juice.
0. Never 1. |__|__| per day
2.|__|__| per week 3. |__|__| per month
88. Don't know **99. No response**

13.7 During the past month, not counting juice, how many times per day, week, or month did you eat fruit? Count fresh or canned fruit.
0. Never 1. |__|__| per day
2. |__|__| per week 3. |__|__| per month
88. Don't know **99. No response**

13.8 During the past month, how many times per day, week, or month did you eat cooked or canned beans, such as baked, black, broad, gungo peas, beans in soup, soybeans, red peas, tofu or lentils. Do NOT include string beans

0. Never	1. |__|__| per day
2. |__|__| per week	3. |__|__| per month
**88. Don't know**	**99. No response**

13.9 During the past month, how many times per day, week, or month did you eat dark green vegetables for example broccoli or dark leafy greens including callaloo, pakchoi, collard greens or spinach?

0. Never	1. |__|__| per day
2. |__|__| per week	3. |__|__| per month
**88. Don't know**	**99. No response**

13.10 During the past month, how many times per day, week, or month did you eat orange- coloured vegetables for example carrots, pumpkin?

0. Never	1. |__|__| per day
2. |__|__| per week	3. |__|__| per month
88. **Don't know**	**99. No response**

13.11 During the past month, how many times per day, week, or month did you eat other vegetables for example lettuce, okra and cabbage?

0. Never	1. |__|__| per day
2. |__|__| per week	3. |__|__| per month
**88. Don't know**	**99. No response**

13.12 During the past month, how many times per day, week, or month did you eat fish for example mackerel, sardine, whole fish, and sliced-fish?

0. Never	1. |__|__| per day
2. |__|__| per week	3. |__|__| per month
**88. Don't know**	**99. No response**

13.13 During the past month, how many times per day, week, or month did you consume dairy or dairy products (not to include condensed milk) for example milk, cheese, flavoured milk, and powdered milk?

0. Never	1. |__|__| per day
2. |__|__| per week	3. |__|__| per month
88. **Don't know**	**99. No response**

13.14 During the past month, how often did you drink regular soda or sugar-sweetened fruits drinks (such as box/ bag 'juice', lemonade and kool-aid) do not include diet beverages?

0. Never	1. |__|__| per day
2. |__|__| per week	3. |__|__| per month

88. Don't know **99. No response**

13.15 Do you usually add salt/salty sauce to your meals at the table?

0. No **(Go to Q13.17)** 1. Yes
88 Don't know (Go to Q13.17) **99 No response (Go to Q13.17)**

13.16 How often is salt/salty sauce/seasonings added in cooking or preparing foods in your household?

0. Never 1. Rarely 2. Sometimes 3 Often 4. Always
88 Don't know **99 No response**

13.17 How often do you eat processed foods high in salt e.g banana chips, canned mixed vegetables, frankfurters?

0. Never 1. Rarely 2. Sometimes 3 Often 4. Always
88 Don't know **99 No response**

From Jamaica Health and Lifestyle Survey
Used with permission

Psychosocial Stress Questionnaire 1

(variable names in parentheses)

ID NO. |___|___|___|___|___|___|___|___|___|___|___|___|___| QUESTIONNAIRE ID NO. [__]__] __] __]
Reg Par Con ED Dw H Re

Date of questionnaire: _ _ / _ _ / _ _ _ _ (dd/mm/year)

Section 1: Acute Life Events

1. **Life time Stress (4 items):**
   For each event, please indicate whether the event occurred at any point in your life:
   a) Death of a child of yours (***deathchild***) [1] Yes [0] No **[99] No response**
   b) Victim of serious physical attack or assault (***attackassault***) [1] Yes [0] No **[99] No response**
   c) Life-threatening illness or accident (***lifethreatself***) [1] Yes [0] No **[99] No response**
   d) Life-threatening illness or accident to spouse or child (***lifethreatfam***) [1] Yes [0] No **[99] No response**

2. **Stress in the past five years (11 items):**
   For each event, please indicate whether the event happened to you in the past five years:
   a) Life-threatening illness or accidental injury to someone else close to you (***lifethreatclose***) [1] Yes [0] No **[99] No response**
   b) Death of someone else close to you (***deathclose***) [1] Yes [0] No **[99] No response**
   c) Involuntarily lost a job for reasons other than retirement (***jobloss***) [1] Yes [0] No **[99] No response**
   d) Being unemployed & looking for work longer than 3 months (***unemployed3m***) [1] Yes [0] No **[99] No response**
   e) Anyone else in your household unemployed & looking for work for longer than 3 months (***unemployed3mother***) [1] Yes [0] No **[99] No response**
   f) Moved to a worse residence or neighborhood (***moveddown***) [1] Yes [0] No **[99] No response**
   g) Being robbed or having home burglarized (***robbed***) [1] Yes [0] No **[99] No response**
   h) Serious financial problems/difficulties (***financialproblems***) [1] Yes [0] No **[99] No response**
   i) Legal trouble (***legalproblems***) [1] Yes [0] No **[99] No response**
   j) Anything else bad happened to you that upset you a lot (***badexperience***) [1] Yes [0] No **[99] No response**
   k) Anything else bad happened to someone close to you that upset you a lot (***badexperienceclose***) [1] Yes [0] No **[99] No response**

Section 2: Employment Related Stress

3. **Job Dissatisfaction (1-item):**

Please look at the respondents answer to Q1.10. Alert the respondent that questions in section 2 must relate to this job. [**For persons who are currently unemployed assign a score of zero (0) and GO TO QUESTION 9**]

a) How satisfied are you with your job? (***jobsatisfaction***) [ ]
(Rate your job with a score ranging from 1-5, **where 1=completely satisfied; 5=not satisfied at all**; **99 = No response**)
[1] [2] [3] [4] [5] [99]

4. **Job Control (3-items; α: 0.71): Interviewer checkpoint: The respondent must refer to the stated job (Q1.10)**
After each statement, please state which of the numbers best match what you think. **1=agree strongly; 4=disagree strongly; 99=no response**: **(circle appropriate number)**
   a) My job requires me to be creative (***jobcreativity***) [1] [2] [3] [4] [99]
   b) My job allows me to make a lot of decisions (**jobdecisions**) [1] [2] [3] [4] [99]
   c) I get to do a variety of different things on my job (**jobvariety**) [1] [2] [3] [4] [99]

5. **Job security** (**2-item mean index, ranging 1-4) (circle appropriate number**)
   a) How likely is it that during the next couple of years you will involuntarily lose your main job? (***losejob*** **reverse coded**; **1 =not at all likely; 4 =very likely; 99 = no response**)
   [1] [2] [3] [4] [99]
   b) If you were to lose your main job, what do you think your chances would be of finding another job that paid the same? (***findjob***)
   Rate your chances **as 'very good', 'good', 'fair' or 'poor' using scale 1 = very good, 4 = poor; 99 = no response** [1] [2] [3] [4] [99]

6. **Work Demands** (**3-item mean index, ranging 1-4):**
After each statement, please tell me the extent to which you agree (**1 = agree strongly; 4 = disagree strongly; 99 = no response**):
   a) I am <u>NOT</u> asked to do an excessive amount of work (***workexcess***)
   [1] [2] [3] [4] [99]

b) I have enough time to get the job done (***worktime***) [1] [2] [3] [4] [99]

c) I am free from conflicting demands that others make (***workconflict***) [1] [2] [3] [4] [99]

7. **Job-Nonjob Conflicts (2-item mean index, ranging 1-4)**
After each statement, please tell me the extent to which you agree or disagree (**reverse-coded so that 1 = disagree strongly ; 4 =agree strongly; 99=no response**):
My job leaves me feeling too tired and stressed after work to

a) Participate in the activities with friends and family that I'd like to (***jobconflict1***)
[1] [2] [3] [4] [99]

b) Participate in the activities in my neighbourhood and community that I'd like to (***jobconflict2***) [1] [2] [3] [4] [99]

8. **Job Hazards (3-item mean index, ranging 1-3)**
For each statement below, please tell me the extent to which you are exposed to dangers on the job. Please rate using the following scale: **1=not exposed; 2=exposed but slight problem not a big issue); 3=exposed and it is a great problem (big issue); 99=no response**

a) Do you have a problem with exposure to dangerous chemicals on your job? (***jobexpos1***)
[1] [2] [3] [99]

b) Do you have a problem with exposure to air pollution from dusts, smoke, gas, fumes, fibers, or other things on your job? (***jobexpos*2**) [1] [2] [3] [99]

c) To what extent does your job expose you to risk of accidents or injury? (***jobexpos*3**)
[1] [2] [3] [99]

Section 3: Financial Stressors

9. **Financial Strain (2-item mean index, ranging 1-5)**

a) How satisfied are you with your/your family's present financial situation? (***financesat)***
Please indicate your level of satisfaction, using a scale of 1-5, where **1= completely satisfied and 5 = not satisfied at all; 99=no response** [1] [2] [3] [4] [5] [99]

b) How difficult is it for you/your family to meet the monthly payments on your bills? (***finacebill***).
Please indicate your level of difficulty, using a scale of 1-5 where **1=not difficult at all, and 5=extremely difficult; 99=no response** [1] [2] [3] [4] [5] [99]

10. **Total Economic Problems (7-item count, ranging 0-7):**
In the last year, which of the following have you done as a result of economic problems to you and your family:

a) Sold possessions or cashed in life insurance to cover day-to-day expenses (***soldpossessions***)
[1] Yes [0] No **[99] No response**

b) Postponed seeing a doctor or other health professional for financial reasons (***postponedoctor***)
[1] Yes [0] No **[99] No response**

c) Been unable to purchase prescribed medications for financial reasons (***notbuymeds***)
[1] Yes [0] No **[99] No response**

d) Borrowed money from friends or relatives to pay for needed everyday expenses (***borrowmoney***)
[1] Yes [0] No **[99] No response**

e) Applied for government assistance through the PATH programme, or other government run social security programmes to help meet everyday expenses (***govassistance***)
[1] Yes [0] No **[99] No response**

f) Obtained a loan to consolidate or pay off debt (***loanfordebt***)
[1] Yes [0] No **[99] No response**

g) Moved to cheaper living quarters or moved in with other people because you could not afford to stay where you were (***cheaperhouse***) [1] Yes [0] No **[99] No response**

Section 4: Discrimination

11. **Past-Year Events (4-item count, ranging 0-4)**
For each item below indicate if any of the following have occurred within the past year

a) Have you ever been unfairly fired from a job or been unfairly denied a promotion? (***fire)***
[1] Yes [0] No **[99] No response**

i. **If yes,** indicate when this last occurred. (***firetime***):
[1] within the last week [2] within last month [3] within last year

b) Have you ever been unfairly <u>not hired</u> for a job? (***nothired***)
[1] Yes [0] No **[99] No response**
   i. **If yes,** indicate when this last occurred. (***nothiretime***):
   [1] within the last week [2] within last month [3] within last year

c) Have you ever been unfairly stopped, searched, questioned, physically threatened or abused by police? (***policeabuse***) [1] Yes [0] No **[99] No response**
   i. **If yes,** indicate when this last occurred. (***policeabusetime***):
   [1] within the last week [2] within last month [3] within last year

d) Have you ever been unfairly prevented from moving into a neighborhood because the landlord or realtor refused to sell or rent you a house or apt? (***housingdiscrim***)
[1] Yes [0] No [**99] No response**
   i. **If yes**, indicate when this last occurred. (***housingdiscrimtime***):
   [1] within the last week [2] within last month [3] within last year

12. **Daily Life Discrimination (5-item mean index, ranging 1-5):**
In your day-to-day life, how often have any of the following things happened to you **(reverse coded)**
Please indicate your answer using the following scale: ***5 = at least once a week; 4 = a few times a month; 3 = a few times a year; 2 = less than once a year; 1 = never;*** **99 = no response**

a) Treated with less courtesy or respect than other people ***(discrim1)***
[1] [2] [3] [4] [5] [99]

b) Received poorer service than others at restaurants or stores ***(discrim3)***
[1] [2] [3] [4] [5] [99]

c) People act as if they think you are not smart ***(discrim4)***
[1] [2] [3] [4] [5] [99]

d) People act as if they are afraid of you ***(discrim5)*** [1] [2] [3] [4] [5] [99]

e) You are threatened or harassed ***(discrim9)*** [1] [2] [3] [4] [5] [99]

13. **Vigilance Against Discrimination (4-item mean index, ranging 1-5):**
In your day-to-day life, how often do you do the following things? **(reverse coded)** *Please indicate your answer using the following scale:* ***5 = at least once a week; 4 = a few times a month; 3 = a few times a year; 2 = less than once a year; 1 = never;*** **99 = no response***:*

a) Prepare for possible insults from other people before leaving home (***vigil2***)
[1] [2] [3] [4] [5] [99]

b) Feel that you always have to be very careful about your appearance (to get good service or avoid being harassed) (***vigil3***) [1] [2] [3] [4] [5] [99]

c) Carefully watch what you say and how you say it (***vigil4***)
[1] [2] [3] [4] [5] [99]

d) Try to avoid certain social situations and places (***vigil6***)
[1] [2] [3] [4] [5] [99]

14. **Job Harassment** (**2-item mean index, ranging 1-5**)
(Reverse coded) *For the items below please indicate your answer using the following scale:* ***5 = at least once a week; 4 = a few times a month; 3 = a few times a year; 2 = less than once a year; 1 = never; 9*****9 = no response***:*

a) How often do your supervisor or coworkers make slurs or jokes about racial or ethnic groups? ***(jobdiscrim4)*** [1] [2] [3] [4] [5] [99]

b) How often do your supervisor or coworkers make slurs or jokes about women? (***jobdiscrim5)***
[1] [2] [3] [4] [5] [99]

15. **Treated Unfairly Job** (**3-item mean index, ranging 1-5**):
How often have you experienced the following situations at work during the last 12 months (**reverse coded**) P*lease indicate your answer using the following scale:* ***5 = at least once a week; 4 = a few times a month; 3 = a few times a year; 2 = less than once a year; 1 = never;*** **99 = no response***:*

a) You have to work twice as hard as others to get the same treatment or evaluation ***(jobdiscrim1)*** [1] [2] [3] [4] [5] [99]

b) Watched more closely than other workers (***jobdiscrim2)***
[1] [2] [3] [4] [5] [99]

c) Unfairly humiliated in front of others at work ***(jobdiscrim3)***
[1] [2] [3] [4] [5] [99]

Section 5: Relationship Stress

16. **Marital Stress: (4-item mean index, ranging 1-5)**

Based on answer from Q1.5 in demographics: ***If no relationship, go to question 18***

For each item below, please indicate your answer using a scale of 1-5 as specified for each question.

a) Taking everything into consideration, how often do you feel bothered or upset by your marriage/relationship? ***(marupset)*** [**coded**: **1=never, 5=very often*;* 99 = no response**]
[1] [2] [3] [4] [5] [99]

b) How much do you feel your partner makes too many demands on you? (***marsup2)*** *[**coded**: **1=not at all, 5=a great deal;*** **99 = no response***]* [1] [2] [3] [4] [5] [99]

c) How much is your partner critical of you or what you do? (***marsup4)*** [**coded**: **1=not at all, 5=great deal*;* 99 = no response**] [1] [2] [3] [4] [5] [99]

d) When you and your partner disagree about something, how often do you work things out so that both of you are satisfied (***marrslve)*** [**reverse coded**: [**1=very often, 5=never*;* 99 = no response**] [1] [2] [3] [4] [5] [99]

Now we're going to talk about somethings that may be hard to talk about. There are no right or wrong answers

17. **Marital Abuse (4-item mean index, ranging 1-4):**

Please indicate how often your spouse/partner engages in the actions below, using a four-point scale, **coded as 1=never, 4=all the time*;* 99 = no response**)

a) Drinks too much (***marbhve1)*** [1] [2] [3] [4] [99]

b) Pushes, slaps, or hits you (***marbhve2)*** [1] [2] [3] [4] [99]

c) Wastes money the family needs for other things (***marbhve3)*** [1] [2] [3] [4] [99]

d) Yells or screams at you (***marbhve5)*** [1] [2] [3] [4] [99]

18. **Child-related Stress (3-item mean index, ranging 1-5)**

*Do you currently have any children? (**childstatus**)*

[1] Yes [0] No **GO TO QUESTION 20** **[99] No response**

a) How much do you feel your children make too many demands on you? Indicate your answer using a scale of 1-5, **where 1=not at all & 5=great deal*; 99 = no response*** (***childdemand)***

[1] [2] [3] [4] [5] [99]

b) How often do you feel bothered or upset as a parent? Indicate your answer using a scale of 1-5, **where 1=never & 5= almost always*; 99 = no response*** ***(botheredp)***

[1] [2] [3] [4] [5] [99]

c) How happy are you with the way your children have turned out at this point? Indicate your answer using a scale of 1-5, **where 1=very happy & 5=not at all happy**) (***parhappy)***

[1] [2] [3] [4] [5] [99]

19. **Total Problems for Children (6-item count, ranging 0-6):**

Please tell me whether any of your children are currently having any of these problems:

a) Problems with finances (***childprob1***) [1] Yes [0] No **[99] No response**

b) Problems with job or trouble finding or keeping employment (***childprob2***)
[1] Yes [0] No **[99] No response**

c) Health problems (***childprob3***) [1] Yes [0] No **[99] No response**

d) Problems with close relationship with others (not counting you or spouse) (***childprob4***)
[1] Yes [0] No **[99] No response**

e) Problems with relationships with you or spouse (***childprob5***)
[1] Yes [0] No **[99] No response**

f) Anything else (***childprob6***) [1] Yes [0] No **[99] No response**

20. ***Friend Criticism (*2 item, mean index, ranging 1-5)**

*Please indicate your answer using a scale of 1-5, **where 1=not at all and 5=a great deal;*** **99 = no response**

a) On average, how much do you feel your friends and relatives make too many demands on you? (***friendsup2)***

[1] [2] [3] [4] [5] [99]

b) How much are they critical of you or what you do (***friendsup4)***

[1] [2] [3] [4] [5] [99]

Section 6: Early Life Stress

Psychosocial Stress Questionnaire 1

(variable names in parentheses)

ID NO. |___|___|___|___|___|___|___|___|___|___|___|___|___| QUESTIONNAIRE ID NO. [__]__] __] __]
Reg Par Con ED Dw H Re

21. **Parental Stress (3-item, mean index, ranging from 1-5)**
   a) Thinking about most of the years until you were 12, how much would you say that your parent(s) (or the adults who raised you) made you feel loved? *Please indicate your answer using a scale of 1-5,* ***where*** **1=great deal & 5-not at all*;* 99 = no response** (***lovedchild***)
   [1] [2] [3] [4] [5] [99]
   b) How much would you say that they physically threatened or abused you? **(reverse coded)** *Please indicate your answer using a scale of 1-5, where* **1= not at all & 5= great deal *;* 99 = no response** (***abusedphysical***) [1] [2] [3] [4] [5] [99]
   c) How much would you say they verbally threatened or abused you? **(reverse coded)** *Please indicate your answer using a scale of 1-5, where* **1= not at all & 5= great deal *;* 99= no response** (***abusedverbal***) [1] [2] [3] [4] [5] [99]

22. **Parental Educational Involvement *(*2 item, mean index, ranging 1-5)**
   Please indicate your answer using a scale of 1-5, where **1=great deal & 5=never; 99 = no response**
   a) Thinking about most of the years until you were 12, how much would you say that your parent(s) or the people who raised you, participated in school activities? (***parrel8)***
   [1] [2] [3] [4] [5] [99]
   b) Did they (your parent(s) or the people who raised you) or other adults read to you? (***parread)***
   [1] [2] [3] [4] [5] [99]

23. **Hunger (1 item)**
   (Reverse coded) *Please indicate your answer using a scale of 1-5,* ***where*** **1=never and 5=very often; 99 = no response**
   a) Thinking about most of the years until you were 12, how often did you go to bed at night feeling hungry? (***hungrychild***) [1] [2] [3] [4] [5] [99]

Section 7: Community Stressors

24. **Violence (5-item, mean index; ranging from 1-4),**
   Please indicate your answer using a scale of 1-4, ***where 1=never, 2=rarely, 3=sometimes, 4=often;*** **99 = no response**
   During past 6 months:
   a) How often was there a fight in your neighborhood in which a weapon was used (***pviol1)***
   [1] [2] [3] [4] [99]
   b) A violent argument between neighbors? (***pviol2)*** [1] [2] [3] [4] [99]

c) Gang fights? (***pviol3)*** [1] [2] [3] [4] [99]
d) Sexual assault or rape? (***pviol4)*** [1] [2] [3] [4] [99]
e) Robbery or mugging? (***pviol5)*** [1] [2] [3] [4] [99]

25. **Total Victimization *(*4-item count, ranging from 0-4):**
While you have lived in your current neighborhood, has/have:
a) Anyone ever used violence, such as in a mugging, fight, or sexual assault against you or any member of your household anywhere in your neighborhood? (***victim1)***
[1] Yes [0] No **[99] No response**
b) Your home ever been broken into? (***victim3)*** [1] Yes [0] No **[99] No response**
c) You or another member of your household had anything stolen from your yard, porch, garage, or elsewhere outside your home but on your property? (***victim5)***
[1] Yes [0] No **[99] No response**
d) You or another member of your household had property damaged, including damage to vehicles parked in the street, to the outside of your home, or to other personal property? (***victim7)*** [1] Yes [0] No **[99] No response**

26. **Disorder (5-item mean index, ranging 1-4)**
Please indicate your answer using a scale of 1-4, ***where 1=none, 2= a little, 3=some, 4=a lot/often;*** **99 = no response**
a) How much broken glass or trash on sidewalks and streets do you see in your neighborhood? (***disord1a)*** [1] [2] [3] [4] [99]
b) How much graffiti do you see on buildings and walls in your neighborhood? ***(disord2a)***
[1] [2] [3] [4] [99]
c) How many vacant or deserted houses or storefronts do you see in your neighborhood? (***disord3a)*** [1] [2] [3] [4] [99]
d) How often do you see people drinking in public places in your neighborhood? (***disord4a)***
[1] [2] [3] [4] [99]
e) How often do you see unsupervised children hanging out on the street in your neighborhood? ***(disord6a)*** [1] [2] [3] [4] [99]

Social Support Questionnaire 1

(variable names in parentheses)

ID NO. |___|___|___|___|___|___|___|___|___|___|___|___|___| QUESTIONNAIRE ID NO. [__]__]__]__]
Reg Par Con ED Dw H Re

Date of questionnaire: _ _ / _ _ / _ _ _ _ (dd/mm/year)

Social Network size

1. How many close friends and relatives do you have (people that you feel at ease with, can talk to about private matters, and can call on for help)? (***friendnumber***)
   Number [ ] **[99] No response**

Instrumental support

2. How many friends and relatives do you have to whom you can turn when you need to borrow something like a household object or a small amount of money or need help with an errand? (***friendloan***)
   Number [ ] **[99] No response**

Informational support

3. How many friends and relatives do you have who you can ask for advice or information? (***friendadvice***)
   Number [ ] **[99] No response**

Informal social integration index

4. How often do you get together with friends, neighbors, or relatives, and do things like go out together or visit in each other's homes? Would you say? (***socialintegration1****)*

   [6] more than once a week
   [5] once a week
   [4] 2 or 3 times a month
   [3] about once a month
   [2] less than once a month
   [1] never
   [99] no response

5. In a typical week, about how often do you talk on the telephone or exchange emails with friends, neighbors, or relatives? Would you say? ***(socialintegration2)***

   [6] more than once a day
   [5] once a day
   [4] 2 or 3 times a week
   [3] about once a week
   [2] less than once a week
   [1] never
   [99] no response

Social Support Questionnaire 2 – Family and Church

(variable names in parentheses)

ID NO. |___|___|___|___|___|___|___|___|___|___|___|___|___| QUESTIONNAIRE ID NO. [__]__]__]__]
Reg Par Con ED Dw H Re

Date of questionnaire: _ _ / _ _ / _ _ _ _ (dd/mm/year)

Section A: Family Social Support

Section A1: Frequency of Support Received and Given

1. How often do people in your family - including children, grandparents, aunts, uncles, in-laws and so on help you out? **Would you say very often, fairly often, not too often, or never**? (***famhelp1***)

   [1] Very often
   [2] Fairly often
   [3] Not too often
   [4] Never
   [5] (*If volunteered*) Never needed help
   [6] (*If volunteered*) I have no family, ***GO TO SECTION B QUESTION 1***
   [99] No response

2. How often do you help out people in your family - including children, grandparents, aunts, uncles, in-laws and so on? **Would you say very often, fairly often, not too often, or never**? (***famhelp2***)

   [1] Very often
   [2] Fairly often
   [3] Not too often
   [4] Never
   [5] (*If volunteered)* Never needed help
   [99] No response

Section A2: Family Network

3. How often do you see, write or talk on the telephone with family or relatives who do not live with you? **Would you say nearly every day, at least once a week, a few times a month, at least once a month, a few times a year, hardly ever or never?** (*famcomm1*)

   [1] Nearly every day (≥4 times a week)
   [2] At least once a week (1 to 3 times)
   [3] A few times a month (2 to 3 times)
   [4] At least once a month
   [5] A few times a year
   [6] Hardly ever
   [7] Never
   [99] No response

   a) ***[New]*** How often do you communicate with your family or relatives who do not live with you on social media (Facebook, WhatsApp, twitter, etc.)? **Would you say nearly every day, at least once a week, a few times a month, at least once a month, a few times a year, hardly ever or never?** (*famcomm2*)

      [1] Nearly every day (≥4 times a week)
      [2] At least once a week (1 to 3 times)
      [3] A few times a month (2 to 3 times)
      [4] At least once a month
      [5] A few times a year
      [6] Hardly ever
      [7] Never
      [99] No response

Social Support Questionnaire 2 – Family and Church
(variable names in parentheses)

ID NO. |___|___|___|___|___|___|___|___|___|___|___|___|___| QUESTIONNAIRE ID NO. [__]__]__]__]
Reg Par Con ED Dw H Re

---

4. How many people in your family would help you out if you needed help? (***famsize***)
[**PROBE**: Could you give me a number?]

   NUMBER [ ] [ ] [99] No response

5. How close <u>do you feel</u> towards your family members? **Would you say very close, fairly close, not too close or not close at all?** (***famclose1***)

   [1] Very close
   [2] Fairly close
   [3] Not too close
   [4] Not close at all
   [99] No response

6. Would you say your family members are very close in their <u>feelings toward each other</u>**? Would you say very close, fairly close, not too close or not close at all?** (***famclose2***)

   [1] Very close
   [2] Fairly close
   [3] Not too close
   [4] Not close at all
   [99] No response

Section A3: Received Emotional Support

7. *Answer question below using a scale of 1-4,* ***where 1 = very often, 2 = fairly often, 3 = not too often, 4 = never;* 99 = No response**

Other than your (spouse/partner), how often do your family members…

a) …make you feel loved and cared for? **Would you say very often, fairly often, not too often, or never?** (***emotionsupport1***) [1] [2] [3] [4] [99]

b) …listen to you talk about your private problems and concerns? (***emotionsupport2***) [1] [2] [3] [4] [99]

c) …express interest and concern in your well-being? (***emotionsupport3***) [1] [2] [3] [4] [99]

Section A4: Negative Interaction

8. *Answer question below using a scale of 1-4,* ***where 1 = very often, 2 = fairly often, 3 = not too often, 4 = never;* 99 = No response**

Other than your (spouse/partner), how often do your family members…

a) …make too many demands on you? (***neg_interact1***) [1] [2] [3] [4] [99]

b) …criticize you and the things you do? (***neg_interact2***) [1] [2] [3] [4] [99]

c) …try to take advantage of you? (***neg_interact3***) [1] [2] [3] [4] [99]

ID NO. |___|___|___|___|___|___|___|___|___|___|___|___|___| QUESTIONNAIRE ID NO. [__]__]__]__]
Reg Par Con ED Dw H Re

Section B: Friend Social Support

1. How often do you see, write or talk on the telephone with your friends? **Would you say nearly every day, at least once a week, a few times a month, at least once a month, a few times a year, hardly ever, or never?** (***friendsupport1***)

[1] Nearly every day (≥ 4 times a week)
[2] At least once a week (1 to 3 times)
[3] A few times a month (2 to 3 times)
[4] At least once a month
[5] A few times a year
[6] Hardly ever
[7] Never
[8] (*If volunteered)* I have no friends *GO TO SECTION C QUESTION 1*
[99] No response

2. How often do your friends help you out? **Would you say very often, fairly often, not too often, or never?** (***friendhelp1***)

[1] Very often
[2] Fairly often
[3] Not too often
[4] Never
[5] (*If volunteered*) Never needed help
[99] No response

3. How often do you help out your friends? **Would you say very often, fairly often, not too often, or never?** (***friendhelp2***)

[1] Very often
[2] Fairly often
[3] Not too often
[4] Never
[5] (*If volunteered*) Never needed help
[99] No response

4. How close do you feel towards your friends? **Would you say very close, fairly close, not too close or not close at all?** (***friendclose***)

[1] Very close
[2] Fairly close
[3] Not too close
[4] Not close at all
[99] No response

Section C: Church / Religious Social Support

Social Support Questionnaire 2 – Family and Church

(variable names in parentheses)

ID NO. |___|___|___|___|___|___|___|___|___|___|___|___|___| QUESTIONNAIRE ID NO. [__]__]__]__]
Reg Par Con ED Dw H Re

Section C1: Religion/Denomination

1. What is your current religion / religious affiliation? (***religion***)

[1] Christian
[2] Rastafarian
[3] Muslim
[4] Other, specify __________________
[5] None
[99] No response

a) **If answer to question C1 above is Christian**, please state denomination / church type (***denomination***)

[1] Catholic
[2] United Church of Jamaica *(Presbyterian, Congregational, Disciples of Christ)*
[3] Seventh Day Adventist
[4] Methodist
[5] Anglican
[6] Baptist
[7] Church of God
[8] Pentecostal
[9] Non-Denominational
[10] Other, specify __________________
[99] No response

Section C2: Organizational Participation

2. Other than for weddings or funerals, have you attended services at a church or other place of worship since you were 18 years old? *[If under 18, ask "Have you attend church or place of worship because of your own choice, i.e. because you wanted to go and not because you were sent/told to go by your parents or guardian]* (***attend_service***)

[1] Yes [0] No **GO TO END OF QUESTIONNAIRE** **[99] No response**

3. How often do you usually attend religious services? **Would you say nearly every day, at least once a week, a few times a month, a few times a year, or less than once a year? (*service_freq*)**

[1] Nearly every day - ≥4 times a week
[2] At least once a week - 1 to 3 times
[3] A few times a month - 1 to 3 times
[4] A few times a year
[5] Less than once a year **GO TO END OF QUESTIONNAIRE**
[99] No response

4. On a typical (Sunday/Saturday) how many hours are you at your church or place of worship? (***service_hours***)

Number of Hours [ ] [ ] HOURS (0-24) **[99] No response**

5. Are you an official member of a church or other place of worship? (***membership***)

[1] Yes [0] No **[99] No response**

---

6. Besides regular service, how often do you take part in other activities at your church within the last year? **Would you say nearly every day, at least once a week, a few times a month, a few times a year, or never?** (***other_activities***)

    [1] Nearly every day - ≥4 times a week
    [2] At least once a week - 1 to 3 times
    [3] A few times a month - 1 to 3 times
    [4] A few times a year
    [5] Never **GO TO C8**
    [99] No response

7. Not including religious services how many hours per week are you at your place of worship? (***nonservice_time***)

    Number of hours [ ] [ ] HOURS **[99] No response**

Section C3: Church / Religious Social Support Network

8. Other than family members, how often do you see, write, or talk on the telephone with members of your church (or place of worship)? **Would you say nearly every day, at least once a week, a few times a month, at least once a month, a few times a year or never?** (***churchcomm1***)

    [1] Nearly every day
    [2] At least once a week
    [3] A few times a month
    [4] At least once a month
    [5] A few times a year
    [6] Never
    [99] No response

9. How many people in your church (or place of worship) do you think would help you out if you needed help? (**PROBE**: Could you give me a number?) (***churchhelp1***)
    NUMBER [ ] [ ] **No response [99]**

10. How often do people in your church (or place of worship) help you out? **Would you say very often, fairly often, not too often, or never?** (***churchhelp2***)

    [1] Very often
    [2] Fairly often
    [3] Not too often
    [4] Never
    [5] (*If volunteered*) Never needed help
    [99] No response

11. How often do you help out people in your church (or place of worship)? **Would you say very often, fairly often, not too often, or never?** (***churchhelp3***)
    [1] Very often
    [2] Fairly often
    [3] Not too often
    [4] Never
    [5] (*If volunteered*) Never needed help
    [99] No response

12. How close are you to the people in your church (or place of worship)? **Would you say very close, fairly close, not too close, or not close at all?** (***churchclose***)

[1] Very close	[3] Not too close
[2] Fairly close	[4] Not close at all
	**[99] No response**

13. How satisfied are you with the quality of the relationships you have with the people in your church (or place of worship)? **Would you say very satisfied, somewhat satisfied, somewhat dissatisfied, or very dissatisfied?** (***church_satisfaction***)

[1] Very satisfied	[3] Somewhat dissatisfied
[2] Somewhat satisfied	[4] Very dissatisfied
	**[99] No response**

Section C4: Received Emotional Support/Negative Interaction

Answer question below using a scale of 1-4, ***where 1 = very often, 2 = fairly often, 3 = not too often, 4 = never;*** **99 = No response**

14. How often do the people in your church…?

a) …make you feel loved and cared for? **Would you say very often, fairly often, not too often, or never?** (***churchsupport1***)	[1]	[2]	[3]	[4]	[99]
b) …listen to you talk about your private problems and concerns? (***churchsupport2***)	[1]	[2]	[3]	[4]	[99]
c) …express interest and concern in your well-being? (***churchsupport3***)	[1]	[2]	[3]	[4]	[99]

15. How often do the people in your church…?

a) …make too many demands on you? (***churchdemands***)	[1]	[2]	[3]	[4]	[99]
b) …criticize you and the things you do? (***churchcriticize***)	[1]	[2]	[3]	[4]	[99]
c) …try to take advantage of you? (***churchexploit***)	[1]	[2]	[3]	[4]	[99]
