# Supplementary material for: Social support and ideal cardiovascular health in urban Jamaica: A cross-sectional study
Source: PLOS Glob Public Health. 2024 Jul 30;4(7):e0003466. doi: 10.1371/journal.pgph.0003466 (PMC11288424; doi:10.1371/journal.pgph.0003466)
Supplement: Attachment — Submitted filename: Response to reviewers comments final.docx [file pgph.0003466.s016.docx]

**Response to Reviewers**

**PGPH-D-23-02025
Social support and ideal cardiovascular health in urban Jamaica: a cross-sectional study
PLOS Global Public Health**

Thank you for a very comprehensive review of our manuscript. We are grateful for all the reviewers’ and editorial comments. We have tried to address all concerns and we are certain the changes we have made as a result have strengthened our manuscript.

Changes in the manuscript are highlighted in yellow for easy recognition.

Please see responses to specific comments below. Responses are in blue font.

Journal Requirements:

1. Please include a complete copy of PLOS’ questionnaire on inclusivity in global research in your revised manuscript. Our policy for research in this area aims to improve transparency in the reporting of research performed outside of researchers’ own country or community. The policy applies to researchers who have travelled to a different country to conduct research, research with Indigenous populations or their lands, and research on cultural artefacts. The questionnaire can also be requested at the journal’s discretion for any other submissions, even if these conditions are not met.  Please find more information on the policy and a link to download a blank copy of the questionnaire here: <https://linklock.titanhq.com/analyse?url=https%3A%2F%2Fjournals.plos.org%2Fglobalpublichealth%2Fs%2Fbest-practices-in-research-reporting.&data=eJxdjcFuwyAQRL_GvnlF7DghBw5tpZ76ExjWQAQsWiDp55dzpXnS6GmkMep2fQi7rZu1-yFnq_o7JMoa0HZ4pjmpy6c501f_-S3lMVeFCYorHgRIeZfrDcRF4r6f9-kq0IZGHHRMOmuHDIbSzKoxvojhRHa9UhbrmP676cq3Vuq0fUzr98iTOmcdK5RIFYjdcC7SoWPpRwzGo47ND1kHB9a2FNamBYN1CXlhrKjZ-FEKcQvZwR-K3FB6> Please upload a completed version of your questionnaire as Supporting Information when you resubmit your manuscript.

**Author’s Response**

Thank you for this recommendation. This document has been completed and an inclusivity statement placed in the manuscript on page 11 lines 304 to 306.

**Journal Requirement**

2.Please provide separate figure files in .tif or .eps format.

**Author’s response**

Thank you for this recommendation. We have formatted the pictures and made them available in .tif format.

**Reviewer Comments:**

**Reviewer: 1**

Thank you for an interesting paper on an important topic. I am happy to see the analysis that was undertaken and I think the findings can be relevant to adding to the literature as well as in policy recommendations. I am including some comments for your consideration.
**Authors’ response:** We are grateful for the very thorough review and insightful comments that you have provided on our manuscript. We have sought to address each comment thoroughly.

Line 59, maybe more appropriate to use the term “social determinants of health”

**Authors’ response:** We have adjusted and used the recommended term. It Is shown in line 57 in the Introduction section.

Line 67-68 the wording is a bit strange, do you mean maintaining treatment targets?

**Author’s Response:** This has been adjusted accordingly to say “..maintaining the recommended levels..” It can be seen in line 65 in the Introduction.

Line 96. This is a long sentence, maybe a colon or split int two?

**Author’s Response:** This has been split into two and is evident in line 97.

Line 107 should be “interact”
**Author’s Response:** This has been corrected and is seen in line 107.

Paragraph starting at line 101 – this section is not very coherent. There are a lot of concepts and arguments at once. From here the introduction could be strengthened. I think a definition of concepts is important as a number of different terms are being used. It is also risky to refer to social networks when this could be confused with online platforms. Maybe fine a term you want to use throughout and stick with it?

**Author’s Response:** We have revised this section to improve clarity and coherence. We have clarified the definitions. This has been clarified in lines 101 to 106. We chose to retain the term social networks, as this is in the social epidemiology literature. Standardized definitions have been referenced.

Line 114 – there is some repetition here. I think a closer look at the wording here is important.

**Author’s Response:** Thank you for bringing this to our attention. We agree, we have removed this sentence completely.

Line 122 – 123. You need to make a better distinction between cardiovascular diseases and what you refer to cardiovascular health. Do you mean cardiometabolic risk factors?

**Author’s Response:** Thank you for this. Cardiovascular Disease (CVD) was defined at the beginning of the introduction lines 53-55; the concept of cardiovascular health, specifically ideal cardiovascular health, is defined in lines 67-71. The wording has been revised to improve clarity.

Line 124 – A statement like this needs references.

**Author response**: We have adjusted this sentence to say “… to our knowledge, no previous research on social support and cardiovascular health has been conducted in Jamaica”, seen in lines 122 to 123.

**Methods**

It would be helpful for readers unfamiliar with Jamaica to know a bit more about this region and these parishes. Are the urban? Rural? Mixed? What kind of populations?

**Author’s Response:** Thank you for bringing this to our attention. We have expanded this section providing details on the parishes studied and the larger study from which this report is derived. We have also provided a map showing the parishes and health regions in Jamica in the appendix to this paper. Changes are shown in lines 131 – 136.

Presumably the data are only self-reported for the first variables you mention? I would suggest separating two sections: self-reported data; objectively measured data. Were any adjustments made to the CCAHS for the Jamaican population? Was this then turned into a scale? How much value is assigned to each question? Perhaps the questions and their score could be moved to a table for quick reference.
**Author’s Response:**

The definitions for the ideal cardiovascular health components require both self-report and measurements. We collected data on doctor-diagnosed hypertension, diabetes, hypercholesterolemia and also measured blood pressure, fasting glucose and fasting cholesterol. That both measurements and self-reports are required for classification, it would be impractical to separate into sections. BMI classification is based on measurements only. Healthy diet, physical activity and smoking status were defined based on questionnaire data. We have revised this section to improve clarity.

Line 154 – Did the blood pressure measurements all take place on the same day/time? Were point-of-care devices calibrated?

**Author’s Response:** Measurements took place in the morning, usually between 7:00 am and 9:00 am. Devices did not require recalibration but were checked regularly to ensure proper functioning. This has been adjusted and explained in lines 176 to 178.

Line 168 – How did you ascertain treatment for hypertension or diabetes?

**Author’s Response:** This has been clarified in lines 192 to 194 to highlight that we used patient reported being diagnosed with hypertension or diabetes by a doctor.

Line 238 – It would be helpful to know what proportion had the five or more components.

**Author’s Response:** This is reported in the results section and is demonstrated in lines 348 to 349 and in Table 2.

Line 273 – typo for repetition. The values described here could be maybe added to the table instead of written out?

**Author’s Response**: Thank you for bringing this to our attention. We have addressed the typo in line 326.

Line 290 – No need to described what is in the table so directly. Maybe just note the main patterns instead.

**Author’s Response:** Thank you for this. We agree and have adjusted the text to reflect shorter statements and only significant differences are expanded on in lines 345 to 347.

I find Table 2 difficult to interpret. I think it would make more sense to show the ICH score by different categories of the values? So BMI categories (<25, 25-30, 30+), Smoking (current, non), Glucose (normal, elevated, diabetes) etc. It would help to get a sense if there is a dose type relationship for some of these variables.

**Author’s Response:** Thank you for this. We have adjusted the table to remove some of the values. We have also attached footnotes to demonstrate reference points of what is normal for each variable seen in lines 371 to 373. Given the study focuses on ideal cardiovascular health, we believe it is best to report the ICH categories.

We have added table looking at these by categories of social support score in the appendix (S3).

I think there may also be too much text that directly describes the table for Table 3 starting from line 306. Maybe summarize the overall findings and trends rather than restating the table.

**Author’s Response**: We have reviewed this, and we agree and as such has truncated the narrative for table 3. This is evident in lines 376 to 387.

**Discussion**

Discussion (line numbers have disappeared)

**Author’s Response:** Thank you for bringing this to our attention. We have addressed this.

You say there is an association with age and ICH-5 but it is an inverse association. This is hardly surprising since people develop more NCDs as they age and perhaps are more likely not to have control than in earlier stages of disease. You need to say something more about these implications of these findings rather than just restating the results.

**Author’s Response:**

We have addressed this by expanding on the other associations and removing age. This is evident in lines 428 to 437.

I think you are ignoring the role that an obesogenic environment plays including access to safe spaces for engaging in physical activity as well as to healthy foods. This has been extensively documented in Jamaica and I would expect to see this as part of the discussion. Alcohol use is also not included here which is high in Jamaica and especially among men. I would think this could also confound some of your findings.

**Author’s Response:** Thank you for this recommendation, we do agree and have addressed the role of the obesogenic environment in our discussion in lines 449 to 460.

There is also something that is not being mentioned which is the impact of mental health on risk behaviors like smoking and eating. This could also factor into sex differences as well as a differential affect across poverty groups. This should also be included.

**Author’s Response**: Thank you for this input. We have expanded on these points and added in the discussion in lines 487 to 492 and 495 to 499.

Implications
You mention neighbourhood characteristics. I did not see this in the paper. There is the overall land value of the area but that is not a built environment or social characteristic and I think this statement is overreaching. This study provides important insight into the role of social support across different SES levels in Jamaica and certainly adds to the literature there. I think you can stick with that.

**Author’s Response:** Thank you, we have adjusted this in lines 516 to 518.

The strengths and limitations should be expanded. There are limitations to self-reported data and the imputation which are not being discussed.

**Author’s Response:** Thank you for this suggestion. We have expanded the strength and limitations section as requested in lines 523 to 541.

**Reviewer #2**

Thank you for providing me with the opportunity to review the paper titled “Social support and ideal cardiovascular health in urban Jamaica: a cross-sectional study”. This paper by Alphanso et. al reports cross-sectional analysis of the association between social support (exposure variable) and ideal cardiovascular health (outcome variable defined as >=5 optimal levels of CVH factors). In general, the data reported, and multivariable logical regression analysis results are fine. However, the paper would benefit from more clarity around the methodological details, sampling strategy, and discussion of key findings, implications of study results, strengths and limitations.

**Author’s Response:** Thank you for reviewing our manuscript and providing valuable inputs to improve the overall readability. We have made every effort to address each comment specifically.

General comments
• The basic underlying assumption in this study was that higher levels of social support leads to improved health behaviors, defined as ideal cardiovascular health (CVH). The authors examined the association between social support and ideal CVH among urban Jamaicans. I have several comments around the study methods.
• First, since none of the study participants achieved Ideal CVH as per the American Heart Association (AHA) guidelines based on 7 metrics, authors selected an arbitrary cut-off, i.e., >=5 levels as optimal CVH. It should be noted as a deviance from the standard definition of ideal CVH and justified in the methods section.

**Author’s Response:** Thank you for this, we have provided a more detail on this in lines 276 - 279. The low prevalence of all seven characteristics has been noted in previous studies and the decision to use the operational definition of 5 or more characteristics was made *a priori*. We have cited our study protocol and a previous publication using this approach.

• Second, Social support was measured using a questionnaire that focused on social network size (number of friends/relatives), instrumental support (it is unclear whether information on the extent, or amount of support was also collected, which might indicate the strength of association by gender), and

information support: were there any questions to assess the quality of information sources? Perhaps, the study tools can be provided in the appendix.

**Author’s Response:** For these analyses we focused on network size only, based on questionnaire items from Viruell-Fuentes and colleagues; we did not address quality of support received. We have included this as a limitation in the discussion point in lines 536 to 538. Other aspects of social support will be explored in future studies. We have now added the study questionnaire in the appendix.

• Third, sample size: 841 participants: 279 males vs 562 females: I’m wondering why a higher proportion of women were sampled in this survey – what was the sampling frame/strategy, how did you ensure that the participants were randomly selected or to ensure a representative sample?

**Author’s Response:** This has been detailed more in the methods section in lines 142 to 153. We used a multi-stage sampling strategy with randomly selected enumeration districts, systematically selected households and on person selected per household. In the analyses we use survey weight to adjust for differences in the sample compared to the population distribution.

• Fourth, the authors found an inverse association for males and positive association between ICH5 and social support among females? What are the factors that explain these findings (sex related differences) needs to be interpreted in the local context – is it females are more influenced by social support in terms of adopting healthy behaviors due to network size, instrumental and informational support (can we say anything about the quality of information support). We need more explanation, possible pathways that link despite having higher access to information and instrumental support, males show lower levels of optimal health behaviors/ideal CVH.

**Author’s Response:** We conducted analyses of the individual ICH factors to identify the drivers of the association. As suggested, this appears to be due the differences in health behaviours, where women with higher levels of social support were more likely to be non-smokers and to have healthy diets, whereas men with higher levels of social support (larger social networks) were more less likely to be non-smokers. We have included reference to this in the discussion (lines 477 to 492) and provided details in the appendix.

• Fifth – how the study results might change in the light of recently introduced new CVH definition by the AHA – addition of 8th metric “Sleep” variable

**Author’s Response:** This is would have been a useful addition; however we do not have data on sleep for this study; we have therefore mention this in our study’s limitations in lines 538 to 539.

• CCAHS: Chicago Community Adult Health Study based social support questionnaire: was it validated for use in Jamaica?

**Author’s Response:** We did not perform formal validation, but pretested the questionnaire to ensure that the questions were appropriate for use in the Jamaican context and participants understand the questions asked. The analyses focused on three questions related to network size reported as numbers. For the analyses we created a social support score by combining the results from the three questions using principal component analysis (PCA). For the PCA we used Kaiser-Meyer-Olkin (KMO) analyses to ensure the appropriateness of PCA for the variables combined. Details are provided in lines 265 to 274.

Abstract:
• Participant’s age – mean, SD (add standard deviation)
**Author’s Response:** Thank you for spotting this oversight. We have added the standard deviation in the abstract in line 40.

Background
• Line: 74 – 78 – slight modification in the way AHA CVH is conceptualized: 4 behavioral risk factors including “smoking status” and 3 biological factors: blood pressure, glucose and cholesterol.

**Author’s Response:** This has been updated and appropriate reference cited in lines 74 to 76.

• Recently AHA added 8th dimension, Essential Life 8 (including sleep variable)
• A mention about the new essential life 8 metrics would be nice to make it more relevant and authors can explain the study was conducted prior to the introduction of CVH 8 metrics.
**Author’s Response:** Thank you for this; this has been addressed in lines 76 to 80.

Methods and Design
• Survey design and sampling strategy requires more clarity

**Author’s Response:** This has been described in more details in lines 142 to 153 and 289 to 294.

• How male and female participants from the same or different households were selected – I understand methods are described in some other paper, but would be useful to know, whether the study adopts simple random sampling or multistage cluster random sampling.
**Author’s Response:** This is now detailed in lines 142 to 153.

What factors might explain skewed distribution of female and male participants included in this survey and analysis.

**Author’s Response:** Locally conducted surveys in Jamaica commonly have more women than men as women are more likely to participate in household surveys. We correct for this using survey weights to ensure that our estimates are representative of the target population. We have provided additional details on this in the methods and discussion.

Survey weights used? Unclear detail

**Author’s Response:** Thank you for this question. Yes, survey weights were used. This has now been explicitly documented in lines 289 to 294.

Sample size calculation – unclear what was the design effect factor used, and why do authors refer to sample size for each phase of the study – line 213.

Author’s Response:

The design effect was used to adjust the sample size to account for the complex survey design.

We have removed the clause “each phase of the study”, as this was not relevant to this paper.

The section has been revised to improve clarity in line 249.

Results
• Report standard deviation (SD) for all mean values. Refer line 261 – 269.

**Author’s Response:** Thank you for bringing this to our attention. We have added the standard deviation to the values in this section, these changes can now be seen in lines 315 to 324 in the Results section.

• FBG and cholesterol values could also be reported in mg/dl for international readers?

**Author’s Response:** Thank you for this suggestion. We have added in the conversion in Table 1 to make it more understandable for international readers.

• Line 273 – delete repetition in “sex differences”

**Author’s Response:** Thank you for pointing this out. We have adjusted the typo and it is now evident in in line 326.

Table 2. Remove commas after each value, either consistently provide all values with 1 decimal point or remove the 2 decimals provided for the total ICH score. Add definition or threshold as footnote in all Tables

**Author’s Response:** Thank you. We have made the necessary adjustments to table 2, removed all decimal places and attached footnotes in lines 369 to 373.

Descriptive analysis – bivariate analysis of social support score by major socio-demographic factors?

**Author’s Response:** Thank you. We have added Table S4 in the appendices to provide these details.

Table 3 – why not show estimates for the overall population, in addition to presenting results stratified by men and women?

**Author’s Response:** Thank you, we have added a new column in Table 3 with estimates for males and females combined. We have also added a column with p-values for sex interaction to justify our sex specific final models.

Discussion
• What explains the association between men living in poorer communities with higher CVH-T5 scores

**Author’s Response**: Thanks for this comment we have now addressed this in the section on gender differences in lines 477-482.

• Gender differences – needs to be explained better – what do we already know from the published literature and particularly from the same region, and influence of social network on CVD and risk factors?

**Author’s Response**: This has been expanded on in lines 477 to 492.

• The last line “the same study, however, documented the lack of approaches to improve social support among social networks” is unclear – what’s the relevance of this statement in terms of contextualizing this study findings.

**Author’s Response**: Thank you for pointing out this ambiguity, we have removed this statement altogether.

• Possible mechanisms – first sentence needs rephrasing or grammatical checks. I would suggest to explain factors linking positive or negative association of social support with gender/sex in the local Jamaican context. Currently it adds little value to our understanding of what might explain differential associations between men and women. Is it possible to run mediation analysis?

**Author’s Response:** Thank you for this observation. To better understand the factors driving the association between social support and ICH and the gender differences observed we reran our models using the seven individual ICH components in lines 411-418 and in Table S8 of the Appendix. The main factors accounting for the differences appear to be related to higher odds of non-smoking and healthy diet among women with high levels of social support and lower odds of non-smoking among men with high social support. This is also addressed in the discussion in lines 487-492.

• Implications – “our review” – can be replaced by Prior studies or published reports as otherwise it is confusing to the readers

**Author’s Response:** Thank you. We have rewritten this section completely.

• The statement “The findings of this study can stimulate the implementation of targeted approaches that factors in socioeconomic conditions of people” is not a direct conclusion from this analysis, as we do not find any significant associations reported between ideal CVH with any of socio-economic factors.

**Author’s Response:** Thank you for pointing this out, we have removed this statement.

• Strengths – authors mentioned that the study data comes from a mix of rural and urban experiences (or residents) – however, in other places the study setting was described as “urban” population.

**Author’s Response:** Thank you for picking up this inconsistency; the study was done in urban communities only. We have clarified this and removed references to rural throughout the manuscript.

• I thought the study estimated sample size required to investigate the association between ideal CVH and social support, then why is the lower sample size a limitation?

**Author’s Response**: Thank you for pointing this out. We have removed small sample size as a limitation.

• Strengths and limitations section – does not highlight issues around the cross-sectional study design, sampling strategy, selection of urban participants and more women sampled, any measurement bias in terms of exposure or outcome assessments or recall bias?

**Author’s Response:** Thank you for this, we have expanded this section to capture these key concepts (lines 523 – 541). Our use of survey weights addressed for the anomaly of having more females than males in our sample. We have also added Tables S2A and S2B in the appendix showing that the weighted age-sex distribution closely resembles the age-sex distribution of the population in urban Jamaica.

Other minor comments
• Figure 4 – add error bars – why not present data for those participants meeting any 1, any 2, any 3 CVH factors and so on.

**Author’s Response:** Thank you for this suggestion. We have added error bars in for the figure, which is now Figure 2 in the revised manuscript.

• Weighted and unweighted Table 1 show different mean age of the study participants – I wonder why there’s a difference in mean age, 47.63 (unweighted mean age), weighted mean age is 38.46

**Author’s Response:** The sample included more older than younger persons as older persons are more likely to participate in surveys. The sample weights correct for the differences in the sample compared to the target population hence to lower mean age. The age sex distribution for the sample, Jamaica’s urban population and the weighted proportions are now shown in Appendix Tables 2A and 2B.

• Appendix 1 – weighted Table. Total cholesterol value 44.24 – seems like a typo error. Please add units and definition to this Table for all laboratory parameters and abbreviations as footnote for BMI, SBP, DBP.

**Author’s Response:** Thank you for highlighting this. It is a typo, we have corrected it, we have also added the units for the included variables and clarified that these are means as standard errors. Abbreviations have been clarified in footnotes.

References:
• 31 looks incomplete,

**Author’s response:** Thank you, this reference is correct, we have validated same, and it is now reference 33. We have added the DOI (https://doi.org/10.3886/ICPSR31142.v1) for easier recognition.

• 44 – why not reference to the original study in Lancet 2017 <https://linklock.titanhq.com/analyse?url=https%3A%2F%2Fwww.thelancet.com%2Fjournals%2Flancet%2Farticle%2FPIIS0140-6736%2816%2931714-7%2Ffulltext&data=eJxdjctqwzAQRb_G3kXoYVvyQou0EAh0UegXqNL4EfRCHsX5_Kp0VxiGw7nDXKunYaZOcOHc-K16p-u5hxQNAVfJI_RBsze7hPf68cp57g8NgeQ1b4QSpaTiE6FMwTgushsouB1T2Y0PJpoVCrEp9EVjgWcqZIGy1iNFytvpv5qqN8R8dOLa8Vub8zwJbuBNtIC_b5p7pFqi8UfDP9_AFNyth0af9_sXZQO9TFJMHVesrVkwyYaLbPFSvUd44Q8uMU2f>

**Author’s response:** Thank you for this suggestion. We have corrected it and referenced the original study by Tawakol published in 2017. This is cited as reference 67.

**Reviewer #3:** This study has explored social support and ideal cardiovascular health which is emerging/novel concept. However, I have added my comments for the more clarity to improve the quality of paper.

Author’s Response: Thank you for taking the time to review our manuscript and for the valuable input you have provided in guiding us to produce a high-quality report. We have taken these comments into careful consideration and addressed each of them individually below.

Abstract: It is written well and concisely within word limits

**Author’s response:** Thank you for this commendation.

Introduction: It is written well, but there is plenty of room for improvement. Keeping subheading in introduction somehow disconnected the two section (CVD and Social networks). The essence of this paper is novelty in intersections between these two concept. Therefore, it is better to remove sub heading of CVD and social supports in background and link the both content concisely, highlight the importance of social supports in CVD health and also indicate the gap in the literature which this study is trying to address. The length of the introduction can be reduced.

**Author’s response:** Thank you, we have removed the subheadings to create a more seamless flow as you recommended. It was a bit difficult to shorten the introduction as other reviewers requested more information.

Methods
Though most of the information/process are presented, it is very difficult to follow the methods of the paper due to following points

- At first, it is not clear whether this study is primary cross-sectional study or researchers did secondary analysis of 2016/2017 Jamaica Health and Lifestyle Survey (JHLS-III) data.

**Author’s response:** The study is a primary cross-sectional study which targeted participants from communities included in The Jamaica Health and Lifestyle Survey III. For this study we collected data on social support and psychosocial stress which were not included in JHLS-III. We also obtained additional community socioeconomic status data from Jamaica’s National Land Agency (line 228) and the Planning Institute of Jamaica (234). We have clarified this in the revised manuscript.

- If it is primary study then what was the Sampling technique. How those 841 sample were selected for the study? Purposive or random sampling method how? This needs clarification

**Author’s response:** The study employed multi-stage sampling technique wherein randomly selected enumeration districts (ED) were used and 20 households per ED were then selected by a systematic sampling approach and thereafter one participant per household selected using the Kish method. This has been further detailed in lines 142 to 153.

- Study population – 15 years and older is included in the study- how was the written informed consent was taken in case on participants age less than 18 years (minor)- ethical aspect. In addition, does ICH component category (BMI and other) defined by AHA comprises the same criteria for the children less than 18 years as well? Please clarify your approach.

**Author’s Response:** Thank you for this question. The study included 15 participants who were 15 – 18 years old at the time of the study. The University of the West Indies Ethics Committee did not require separate written parental consent for participants older than 15 years and therefore formal parental consent was not obtained. The participants therefore provided written informed consent with approval of their parents or guardians.

Except for physical activity we used the same cut-points for persons 15-19 years old and those 20 years and older and using the percentile cut-points would result in higher cut-off points for those 15-19 years compared to adults. Details are provided in lines 212 -217.

- Sample size: Total number of calculated sample size is very confusing. Looking at abstract and line 210-211 of sample size calculation section of methods, it is evident that sample size of the study is 841. However, line 217-223 makes it more confusing indicating “effective sample size was estimated to 324”. In addition, author has indicated ref no 11,30 as previously published method paper of this study where total number of sample size does not match with the current study. Please justify your sample size approach clearly to avoid confusion to the reader.

**Author’s Response**: We have adjusted the sample size section to provide further clarity. The effective sample size is the actual sample size divided by the design effect (i.e. 841/2.6 = 324) to account for the multi-stage sampling and the use of survey weights. We have provided a reference to the use of a design effect in adjusting sample size estimates.

- In addition, please also assure that this is not the salami slicing publication from the ref no 30., if not how?

**Author’s Response:** Reference 30, now 3, is the study protocol which outlined the plans for the study. The protocol contains no results. This manuscript addresses objective 3 from the study protocol.

- There are plenty of typo errors such as in line 273.. sex differences ( double)

**Author’s Response:** Thank you, we have corrected this.

Result: Result presented in the text is repetition of what is included in the tables. Please try to highlight important findings of the table in the text rather than describing all variables/findings. Present result concisely and clearly.

**Author’s Response:** Thank you for this, we have adjusted this to summarize the results in the text.

Discussion:
- Author has separated this section with sub heading (main findings, comparison to previous study, gender differences and possible mechanisms), however it would be better if it can be written by highlighting important findings and comparing with previous literature with possible explanations together rather than separately. Linking of the information between the paragraph or flow of the information is missing in discussion section, though content is there. Need more evidence to support argument presented in the discussion especially in the possible

**Author’s Response:** Thank you, we have removed the subheadings and have provided more evidence to support our key points. We have also synthesized the sections more to provide comparison with other studies.

mechanisms sections.
- There are plenty of typos and grammatical errors such as second para of discussion ( line no not provided) “protective effectives of social support “… Similarly in implications sections … “sex specific approaches to achieving ICH characteristics” and sentence structure are not clear in possible mechanisms section. English editing needed.
Best wishes

**Author’s Response:** Thank you for pointing these out. We have reread and edited the manuscript to remove typos and grammatical errors.
